# Skin Microbiota Was Altered in Crocodile Lizards (*Shinisaurus crocodilurus*) With Skin Ulcer

**DOI:** 10.3389/fvets.2022.817490

**Published:** 2022-02-14

**Authors:** Haiying Jiang, Shuyi Luo, Jiabin Zhou, Wenzhong Huang, Linmiao Li, Xiujuan Zhang, Jiasong He, Jinping Chen

**Affiliations:** ^1^Guangdong Key Laboratory of Animal Conservation and Resource Utilization, Guangdong Public Laboratory of Wild Animal Conservation and Utilization, Institute of Zoology, Guangdong Academy of Sciences, Guangzhou, China; ^2^Guangxi Daguishan Crocodile Lizard National Nature Reserve, Hezhou, China

**Keywords:** crocodile lizard, dermatosis, skin microbiome, conservation, endangered animal

## Abstract

Skin diseases commonly affect reptiles, but their relationships to the closely related skin microbiome are not well-understood. In recent years, both the wild and captive populations of the crocodile lizard, a Class I protected endangered animal in China, have suffered serious skin diseases that hamper the rescue and release projects for their conservation. This study conducted a detailed prevalence investigation of a major dermatosis characterized by foot skin ulcer in crocodile lizards. It should be noticed that skin ulcer has been prevalent in both captive and wild populations. There was positive correlation between skin ulcer and temperature, while no significant relationship between skin ulcer and humidity, sex, and age. We further studied the relationship between skin ulcer and the skin microbiota using meta-taxonomics. Results showed that the skin microbiota of crocodile lizards was significantly different from those of the environmental microbial communities, and that skin microbiota had a significant relationship with skin ulcer despite the impact of environment. Both bacterial and fungal communities on the ulcerated skin were significantly changed, which was characterized by lower community diversity and different dominant microbes. Our findings provide an insight into the relationship between skin microbiota and skin disease in reptile, serving as a reference for dermatological etiology in wildlife conservation.

## Introduction

Recent studies have shown that microbiome dysbiosis is an important factor in many diseases. The dysbiosis of the gut microbiota has been related to infection, autoimmunity and autoinflammation, and metabolic syndrome (1). The dysbiosis of oral microbial community is associated with dental caries and periodontitis (2), whereas a disrupted vaginal microbiota is associated with bacterial vaginosis (3). Further, changes in skin microbiota are related to skin diseases such as acne and atopic dermatitis (4).

In conservation biology, the potential role of the microbiome on animal conservation is receiving increasing attention, including host health and disease, habitat degradation and nutrition utilization (5, 6). However, most studies have focused on the gut microbiome, and relatively few studied the microbes on the skin.

Skin microbes have been recognized to play an important role in health. The skin microbiota interact with the host and modulate the host's gene expression (7). Once immunity is compromised, the normal bacteria on the skin may turn pathogenic (8). The dysbiosis of skin microbiota dynamics is associated with many skin diseases, such as atopic dermatitis and allergic skin diseases in humans and companion animals, bovine digital dermatitis, demodectic mange, bat white nose syndrome, and camel dermatophilosis (4, 8). Moreover, the perturbations in the skin microbiota are dependent on disease severity in some skin diseases (9). In amphibians, pathogen infection altered the normal skin microbiota (10). Conversely, it has been shown that the normal microbiota can resist diseases; altering the microbial interactions on frog skins can prevent a lethal disease outcome (11). The skin microbiota of red-backed salamander (*Plethodon cinereus*) can reduce *Batrachochytrium dendrobatidis* (Bd) infection, and the resistance is influenced by temperature, which directly impacts pathogen load, and thus has an indirect effect on the changes in the skin microbiome and host mortality (12). Moreover, probiotic-aimed strategies, which aim to increase the diversity and the abundance of beneficial bacteria of the skin microbiota, have become a focal point in the treatment of skin diseases and dysbiosis in humans and amphibians (5, 13–15).

Despite the fact that dermatologic disease is one of the most common diseases in reptiles, only a few studies have been reported about the reptilian skin microbiome (16–18). Most studies on the skin microbiome focus on humans, companion animals, domestic animals, and amphibians, with only a few investigations on fish and birds (8).

Crocodile lizard (*Shinisaurus crocodilurus*) is a monotypic species of the family Shinisauridae and is described as a living fossil reptile. It is an endangered lizard worldwide (19), an Appendix I species of the Convention on International Trade in Endangered Species of Wild Fauna and Flora (CITES I), and a national first-class protected animal in China. The total number of crocodile lizards in the wild is estimated to be 1167–1325, including 1020–1178 in China (Chinese data from the communication on the 2nd International Symposium on Crocodile Lizard) and 147 in Vietnam (20). Their wild population is smaller than that of the giant panda, a flagship species.

Therefore, several nature reserves are conducting captive breeding and release programs to save this species. There are ~800 captive crocodile lizards globally. However, in recent years, they have been affected by various diseases, such as cutaneous granuloma, skin ulceration, early death in newborns, malformation, incomplete absorption of yolk, and seizure (21–23), thus impeding conservation programs and increasing the risk of transmission to the wild population. Among these, the most widespread and serious are skin diseases. Particularly, foot skin ulcer is prevalent both in the wild and captive populations, occurring every year and causing many deaths in crocodile lizards in captivity (21). Its causative agent remains unknown.

Several studies have shown that the gut microbiome can modulate skin diseases, such as atopic dermatitis, through the gut-skin axis (24). However, we have previously showed that skin ulcer was not significantly related to the gut microbiota in crocodile lizards (21). Therefore, in this study, we focused on elucidating the relationship between the skin microbiota and skin diseases, providing foundation for conservation policies and strategies to prevent species extinction due to skin diseases.

## Materials and Methods

### Prevalence Investigation

Prevalence investigation of skin diseases in wild crocodile lizards was conducted in the Guangxi Daguishan Crocodile Lizard National Nature Reserve, including the Chishui Chong, Dachai Chong, and Yusan Chong streams from 2017 to 2019. The wild crocodile lizards were caught at night when they appeared to be sleeping on bush branches near the streams. After checking their health status and identifying the type of diseases, the crocodile lizards were released *in situ*. Prevalence was determined using the equation: P = N_s_ (Lizards with skin ulcer disease)/N_t_ (Total lizards examined).

The prevalence of skin ulcer in the captive crocodile lizards in the Guangxi Daguishan Crocodile Lizard National Nature Reserve was continuously tracked in 2017–2019. Daily temperature and humidity in the artificial simulation ecological pools (the places for breeding the captive crocodile lizards) were recorded every 2 h using Datalogger L99-LXWS (Hangzhou Luge Technology Co. LTD, China). The correlation between the prevalence and the daily mean temperature or humidity were calculated using the Spearman's correlation analysis. At each survey date, the differences in prevalence between groups [female vs. male; adults (> 2 years-old) vs. subadults (1–2 years-old)] were tested by Pearson's chi-squared test or Fisher's exact test.

Average mortality in 2017–2019 was determined using the equation: R =N_d_ (Lizards died with skin ulcer disease)/N_s_ (Lizards with skin ulcer disease).

### Sample Collection in Microbial Analyses

All samples were collected from Beilou station in the Guangxi Daguishan Crocodile Lizard National Nature Reserve on May 19^th^ in 2017. Seven healthy and nine sick crocodile lizards were collected for microbial analyses. Sterile swabs were used to collect the microbes on ulcerative skin on the feet of sick crocodile lizards, as well as on the corresponding sites of healthy individuals. Water and soil samples from wild streams and feeding ponds were also collected to identify the microbes in habitat of crocodile lizards. For the water samples, the solid precipitate was collected using vacuum filtration with 0.2 μm filter membrane. The 28 samples collected were divided into 4 groups, namely, the ulcerative skin group (Sick, *n* = 9), healthy skin group (Healthy, *n* = 7), water group (Water, *n* = 6), and soil group (Soil, *n* = 6). All samples were stored in RNA-EZ Reagents RNA-Be-Locker A [Sangon Biotech (Shanghai) Co., Ltd., China] and transported with ice to the lab for DNA extraction.

### DNA Extraction and Sequencing

DNA from the skin samples were extracted using a QIAGEN DNeasy^®^ Blood & Tissue Kit (QIAGEN China (Shanghai) Co., Ltd.) with 20 mg/mL lysozyme. Because the type of pathogen that causes this disease is still unknown, we used next-generation sequencing to detect differences in both bacteria and fungi between healthy and sick crocodile lizards. For the bacterial community analysis, the V3–V4 hypervariable regions of the 16S rRNA gene were amplified using the primers 341F (5′-CCTAYGGGRBGCASCAG-3′) and 806R (5′-GGACTACNNGGGTATCTAAT-3′). For the fungal community analysis, the *ITS1* gene was amplified using the primers ITS5-1737F (5′-GGAAGTAAAAGTCGTA ACAAGG-3′) and ITS2-2043R (5′-GCTGCGTTCTTCATCGATGC-3′). The amplicon library was prepared using a TruSeq^®^ DNA PCR-Free Sample Preparation Kit (Illumina, Inc., America), and sequencing on Illumina HiSeq2500 platform (paired-end 250 bp) was performed by the Novogene Corporation (China).

To verify the reproducibility of the results of skin microbiota, we collected another 12 samples (5 healthy and 7 ulcerative skin samples) at Gandong station in the Guangxi Daguishan Crocodile Lizard National Nature Reserve in 2020. The whole sequences of 16S rRNA and ITS genes of these 12 samples were amplified using the primers 27F (5′-AGRGTTTGATYNTGGCTCAG-3′) and 1492R (5′-TASGGHTACCTTGTTASGACTT-3′), ITS1 (5′-CTTGGTCATTTAGAGGAAGTAA-3′) and ITS4 (5′- TCCTCCGCTTATTGATATGC-3′), respectively. Sequencing was performed on PacBio platform by the Biomarker Technologies Corporation (China). Figures of this sequencing result could be found in the supporting materials (Supporting Information 2).

### Sequence Analysis

Raw sequences were filtered using QIIME 1.7.0 package (25) to remove the low-quality sequences and chimeras. Sequences with ≥ 97% similarity were assigned to the same operational taxonomic units (OTUs) using Uparse (26). The reads couldn't be clustered to OTUs were removed in the subsequent analysis. Bacterial OTUs were annotated using sequence alignment based on the SILVA database (27) using the Mothur software (28) with threshold 0.8. Fungal OTUs were annotated using sequence alignment based on the UNITE database (29). For comparison among the groups, the OTU abundances were normalized with the number of DNA sequences obtained from the sample with the lowest counts. Abundances of each taxon between the groups were compared by *t*-test if the data conform to the normal distribution and homogeneity of variance.

Alpha diversity was estimated using the Shannon index that was calculated using QIIME 1.7.0 (25). Alpha diversity index was compared among the groups using Wilcoxon test. Meanwhile, beta diversity was analyzed using principal coordinate analysis (PCoA) on unweighted and weighted UniFrac distances. The unweighted and weighted UniFrac distances were calculated using QIIME 1.7.0 (25). In addition, the unweighted pair-group method with arithmetic means (UPGMA) clustering was performed using QIIME 1.7.0 (25). Permutational multivariate analysis of variance (PERMANOVA) statistical analyses were conducted based on the unweighted and weighted UniFrac distances, respectively, with 999 permutations using adonis function in the R package “vegan” (30).

Function prediction of skin bacteria was conducted using PICRUSt (http://picrust.github.com/picrust/) based on the KEGG Orthologs. Principal components analysis (PCA) of bacterial function was conducted using QIIME 1.7.0 (25).

To identify the potential pathogens accounting for skin ulceration, the linear discriminatory analysis (LDA) effect size (LEfSe) method was used to screen the taxa with differential bacterial abundances among groups. LEfSe analysis was performed using the online version of LEfSe software (http://huttenhower.sph.harvard.edu/galaxy/) with threshold of *P* = 0.05 for the Kruskal–Wallis test among the groups. Only those taxa whose log LDA score > 4 were considered in this study. In addition, Venn diagram was used to screen for bacteria or fungi that were shared or unique to the ulcerated skin.

### Bacterial Isolation and Cultivation

Ulcerated skin was spread on a lysogeny broth (LB) agar plate. The plate was incubated at 30°C for 24 h, after which the bacteria were isolated and purified using repeated plate streaking. Bacterial DNA was extracted using a TIANamp Bacteria DNA Kit DP302 (Tiangen Biotech (Beijing) Co., Ltd). The 16S rDNA gene was amplified using the universal primers 27Fs (5′-GAAGTCATCATGACCGTTCTGCAAGAGTTTGATC MTGGCTCAG-3′) and 1492Rs (5′-AGCAGGGTACGGATGTGCGAGCCTACGGH TACCTTGTTACGACTT-3′) and sequenced using 1S (5′-GAAGTCATCATGACCGT TCTGCA-3′) and 2RS (5′-AGCAGGGTACGGATGTGCGAGCC-3′). The bacteria were annotated using sequence alignment based on the NCBI database in BlastN (https://blast.ncbi.nlm.nih.gov).

### Antibiotic Sensitivity Test for Isolated Bacteria

Antibiotic sensitivity test was conducted using the disk diffusion method (31). The purified bacteria were spread on the Mueller–Hinton broth (MH) agar plates. Then disks containing minocycline (30 μg), levofloxacin (5 μg), ampicillin (10 μg), rifampicin (5 μg), kanamycin (30 μg), gentamicin (10 μg), streptomycin (10 μg), cefoxitin (30 μg), erythromycin (15 μg), clarithromycin (15 μg), ciprofloxacin (5 μg), piperacillin (100 μg) (Hangzhou Microbial Reagent Co., LTD., China) were placed on the agar surface. The plates were incubated for 18–24 h at 30°C. The inhibition zone of each antibiotic was recorded.

## Results

### Case Description and Prevalence Investigation

**Figure 1:**
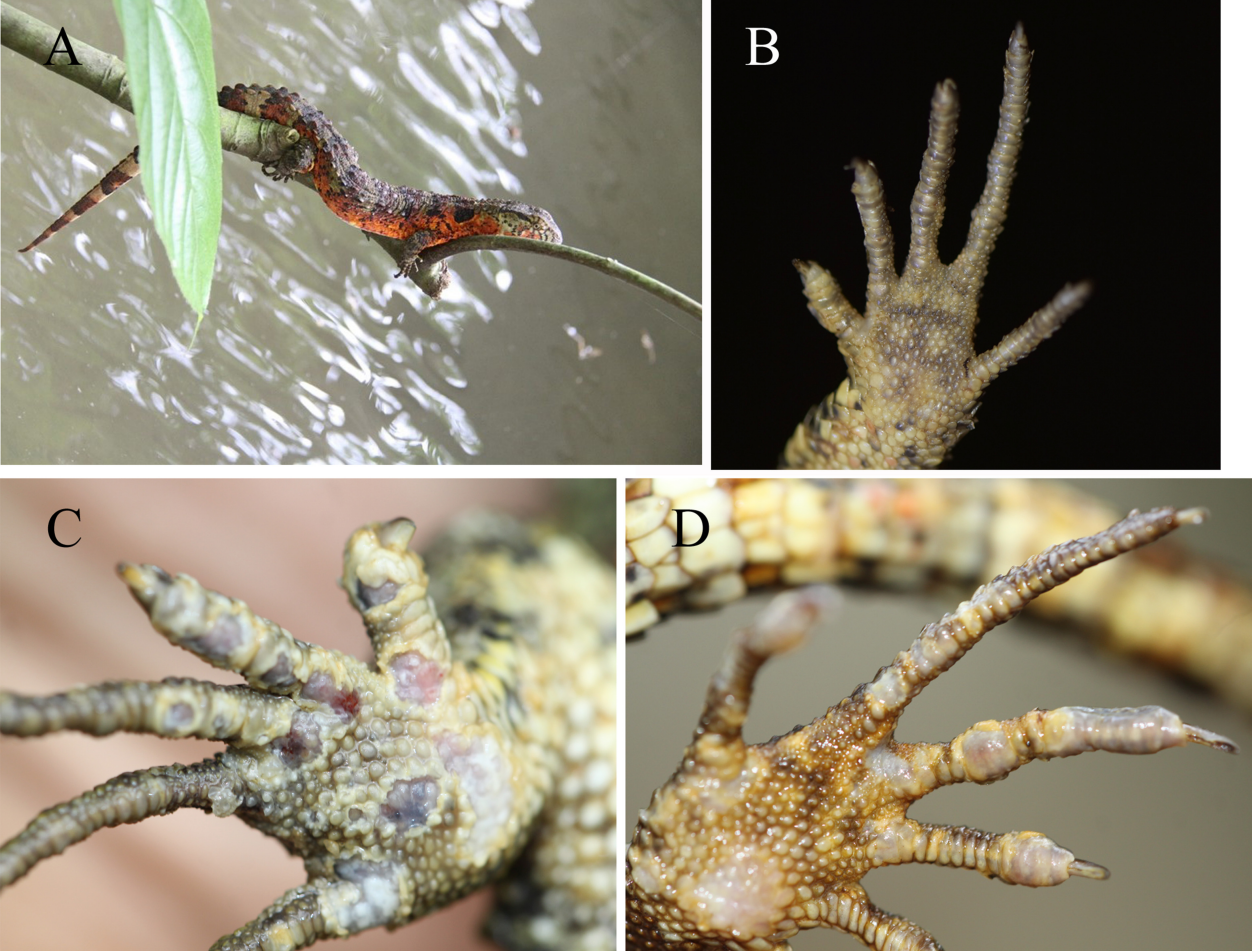
Foot comparison of healthy and sick crocodile lizards. Healthy crocodile lizard **(A)**. Healthy foot **(B)**. Sick foot **(C,D)**. Sick crocodile lizards were afflicted with skin ulceration.

**Figure 2:**
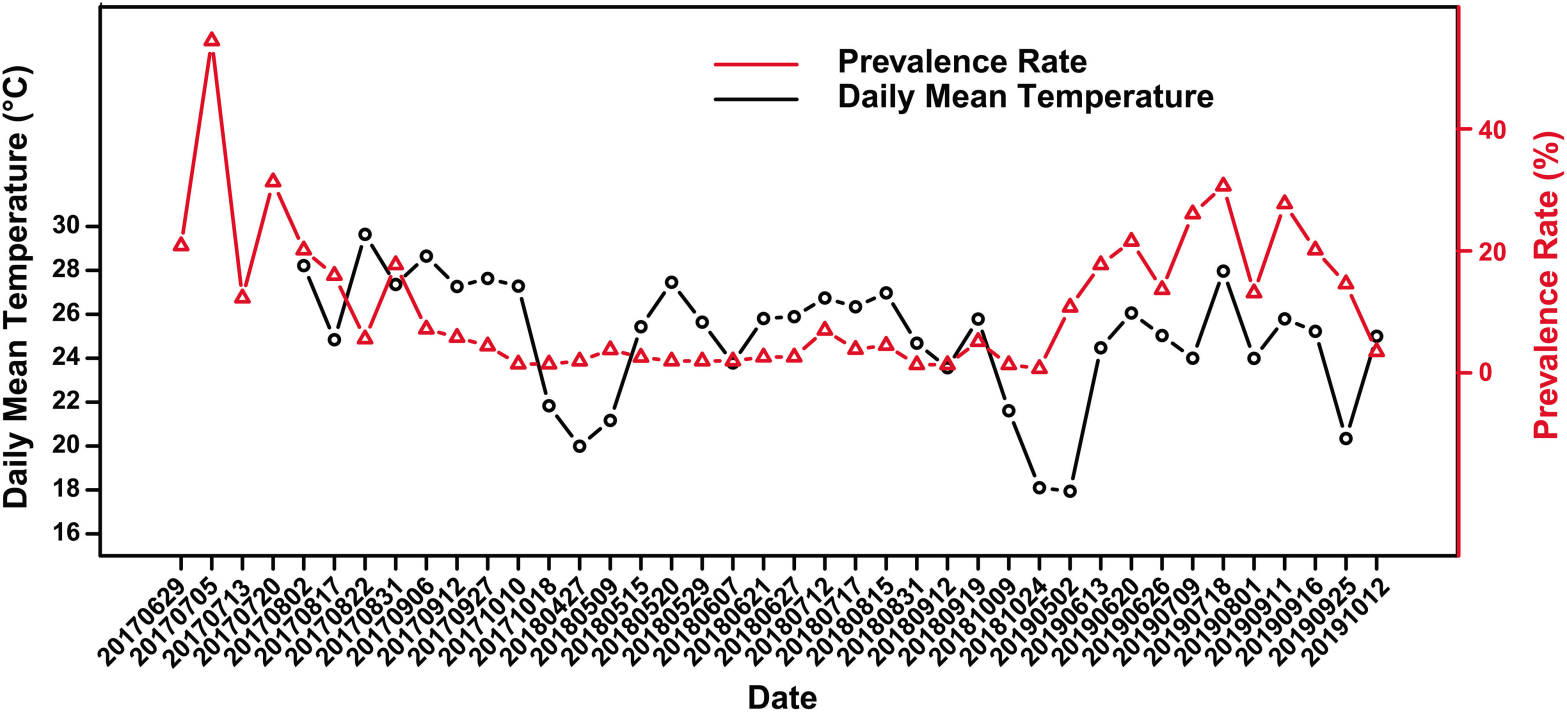
Variation of skin ulcer prevalence in captive crocodile lizards and the daily mean temperature.

The crocodile lizards with ulcerated skin have been observed in Guangxi Daguishan Crocodile Lizard National Nature Reserve and Guangdong Luokeng *Shinisaurus crocodilurus* National Nature Reserve. The main symptoms described on the skin on the feet were ulceration and swelling (Figure 1). The mass was hard and fluid free. This disease has been found in both the wild and captive populations. In Guangxi Daguishan Crocodile Lizard National Nature Reserve, the prevalence of skin ulcer in the captive population was up to 53.75% on July 5th, 2017 (Figure 2), whereas in the wild population, the prevalence varied in different streams (Table 1). The wild crocodile lizards in Chishui Chong stream were in the best health, and no diseased crocodile lizard was found here in 2018–2019. Meanwhile, in the Dachai Chong stream, one case of skin ulcer was recorded each in 2018 (2.17%) and 2019 (1.72%). The prevalence of skin ulcer in Yusan Chong stream was relatively high, ranging from 3.23 to 10%, and skin ulcer cases were observed yearly in 2017–2019.

**Table 1:** Prevalence of skin ulcer in wild crocodile lizards from various streams.

**Investigation date**	**Prevalence (Crocodile lizards with skin ulcer/Total crocodile lizards investigated)**		
	**Chishui Chong Stream**	**Dachai Chong Stream**	**Yusan Chong Stream**
2019.09	0/23	0/50	0/17
2019.08	NA	0/73	**1/17 (5.88%)**
2019.07	0/34	**1/58 (1.72%)**	**2/20 (10.00%)**
2018.09	0/17	**1/46 (2.17%)**	0/21
2018.08	0/16	0/33	**1/31 (3.23%)**
2018.07	0/22	0/36	**1/30 (3.33%)**
2017.05	NA	NA	**2/21 (9.52%)**

*NA, Not available. Case of crocodile lizards with skin ulcer were highlighted in bold. The prevalence was showed in parentheses*.

Skin ulcer was first observed in 2012 and caused many deaths yearly. The mortality rate declined in recent years through the isolation of sick individuals and disinfection. However, in Guangxi Daguishan Crocodile Lizard National Nature Reserve, the captive population's average mortality was 5.52% in 2017–2019.

Skin ulcer is generally more severe in summer than winter. Overall, there was a weak correlation between prevalence and the daily mean temperature according to the scatter plot (Figure 2) and Spearman's correlation analysis (*r* = 0.299), with a significant positive correlation in 2018 and 2019 (*r*_2018_ = 0.638, *P*_2018_ = 0.008; *r*_2019_ = 0.655 *P*_2019_ = 0.034). Further, there was no significant difference in prevalence between female and male (Supplementary Table 1). However, the significance of difference in prevalence between adults and subadults were unstable. Sometimes the prevalence of subadults was significantly higher than that of adults, and sometimes it was not (Supplementary Table 2).

### Sequencing Data Assessment

Twenty-eight samples were sequenced for the bacterial community analysis, including 9 ulcerated and 7 healthy skin samples, and 6 water and 6 soil samples. After quality control, each sample contained at least 37,313 effective sequences for OTU analysis, and the rarefaction curve showed that the sequencing depths were enough to capture most bacterial OTUs (Supplementary Figure 1A). In total, 9,227 bacterial OTUs and 447 archaeal OTUs were obtained in all samples. These OTUs were annotated into 46 phyla of bacteria and 8 phyla of archaea. For the crocodile lizards, 5,385 bacterial OTUs and 57 archaeal OTUs were obtained.

For the fungal community analysis, 16 samples were sequenced, including 9 ulcerated and 7 healthy skin samples. After quality control, each sample contains at least 48,908 effective sequences for OTU analysis, and the rarefaction curve showed that the sequencing depths were enough to capture most fungal OTUs (Supplementary Figure 1B). In total, 3,586 fungal OTUs were obtained in crocodile lizards, which were annotated into 7 phyla.

### The Healthy Skin Microbiota Was Different From the Environment and Was Dynamic

**Figure 3:**
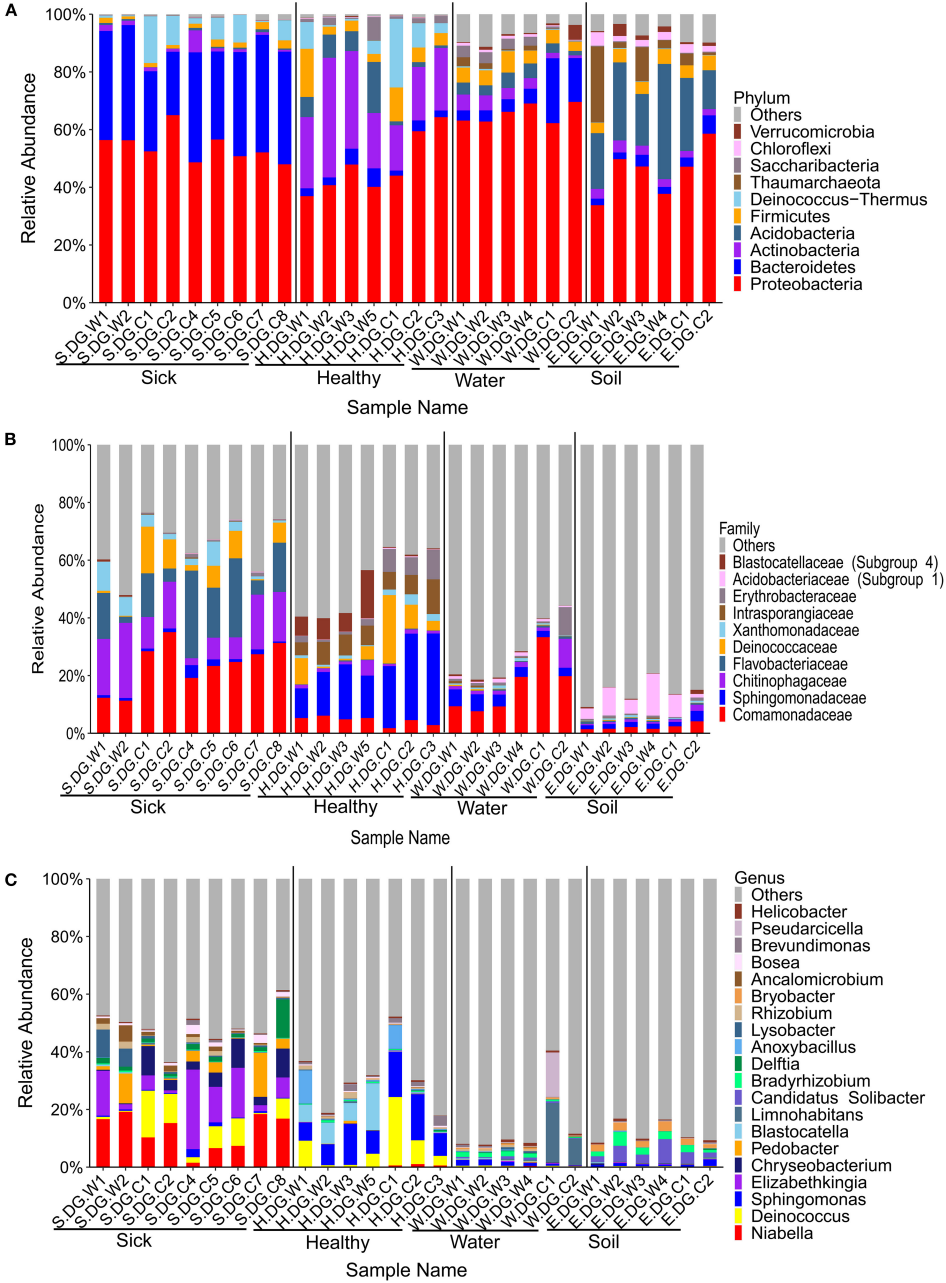
Composition of cutaneous bacteria at the phylum **(A)**, family **(B)**, and genus **(C)** levels. The taxa with abundance of top 10, 10, 20 were showed at the phylum, family, and genus levels. The rest taxa with low abundance were included in “Others.” Group boundaries were separated by black vertical lines.

**Figure 4:**
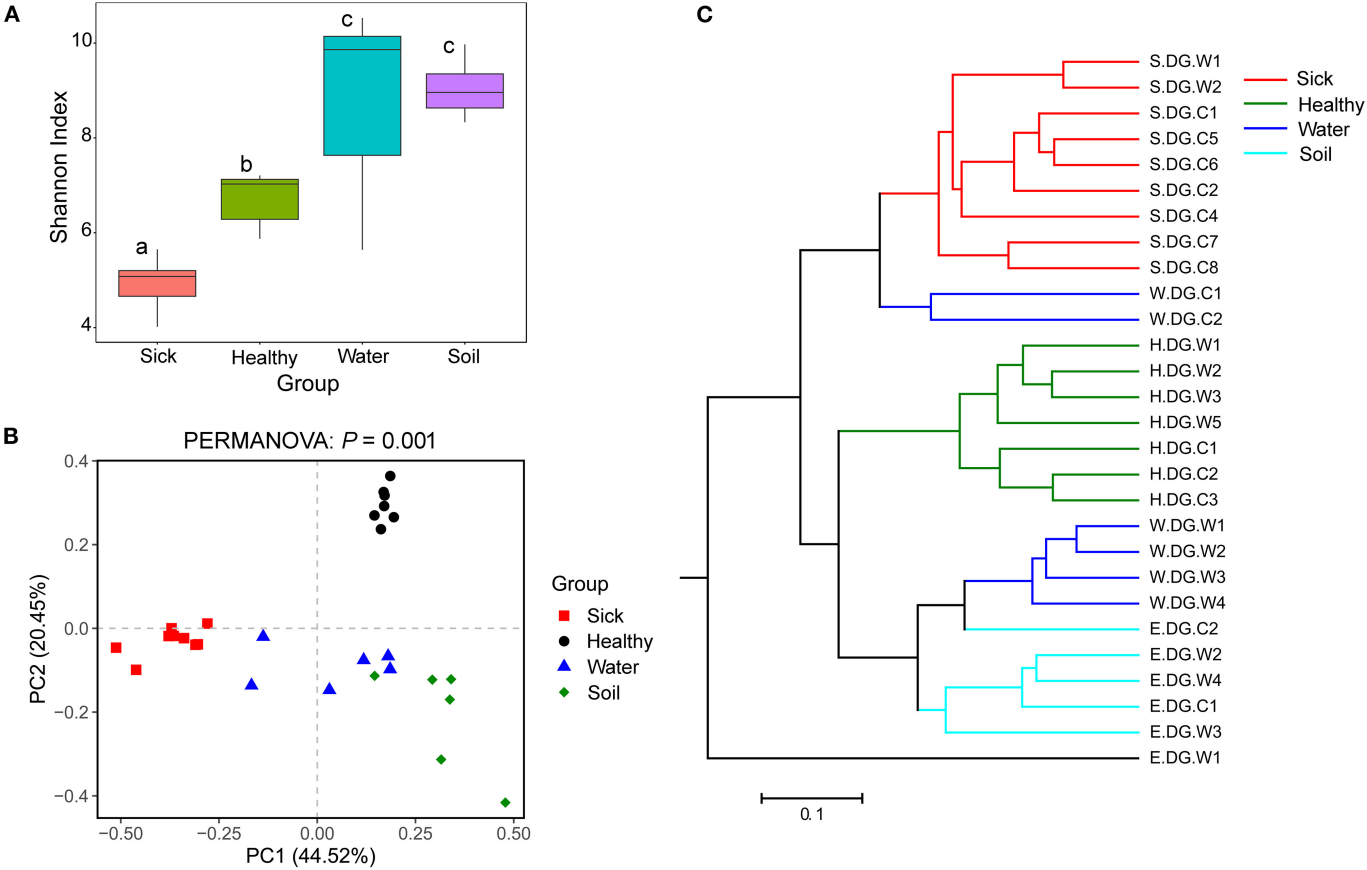
Community diversity of the cutaneous bacterial microbiome. **(A)** Alpha diversity indicated by Shannon index. Data with different superscript letters are significantly different (*P* < 0.05) according to Wilcoxon test. **(B)** Beta diversity indicated by principal coordinate analysis (PCoA) and based on the weighted UniFrac distance matrix. Numbers inside the parenthesis in the axis label show the percentage variation explained by each PC. *P*-value of PERMANOVA test is noted at the top of PCoA plot. **(C)** Beta diversity indicated by the UPGMA cluster and based on the weighted UniFrac distance matrix.

The community composition and the diversity of healthy foot skin microbiota of crocodile lizard was significantly different with those of the environmental water and soil (Figures 3, 4). Comparison of two sequencing results in 2017 (Figures 3, 4, **6**, **7**) and 2020 (Supplementary Figures 5, 7–9) showed that the composition of skin microbiota of healthy crocodile lizard was dynamic. The most abundant bacterial phylum on the foot skin was relatively stable, and was Proteobacteria (> 40%). The other dominant bacterial phyla include Actinobacteria, Firmicutes, Acidobacteria, and Bacteroidetes, while their abundance varied greatly between two experiments. For the fungal analysis, the most abundant at the phylum level was also stable, and was Ascomycota (> 50%). The greatest variation of skin microbiota was at the level of family, genus, and OTU.

### Skin Ulcer Altered the Bacterial Community Structure of the Skin

The bacterial composition of ulcerated skin was significantly changed compared with the healthy skin (Figure 3). At the phylum level, the bacterial community of the ulcerated skin was dominated by Proteobacteria and Bacteroidetes, which accounted for 88.70% of the total bacteria, and was significantly higher than that of healthy group (*P* < 0.001). At the same time, the relative abundances of Actinobacteria and Acidobacteria (the dominant bacteria in healthy skin) were significantly reduced in the ulcerated skin (*P*_Actinobacteria_ < 0.001, *P*_Acidobacteria_ = 0.044). At the genus level, the dominant genera *Niabella, Elizabethkingia*, and *Chryseobacterium* were significantly increased while *Sphingomonas* were notably decreased in the ulcerated skin (*P*_*Niabella*_ < 0.001, *P*_*Elizabethkingia*_ = 0.011, *P*_*Chryseobacterium*_ = 0.007, *P*_*Sphingomonas*_ = 0.001). The change in bacterial composition was also verified by the sequencing results from Gandong station in 2020 (Supplementary Figures 5, 6). Overall, 74 bacterial strains were isolated and purified from the ulcerated skin and were annotated into 11 species. Consistent with the sequencing results, *Elizabethkingia miricola* had the highest abundance (55.41%) (Supplementary Figure 2). The broad-spectrum antibiotics minocycline and levofloxacin were used to treat the ulcerated skin in the ecological simulation pool because it has exhibited an inhibitory effect on all the bacteria isolated from the ulcerated skin (Supplementary Table 3). However, their effect was limited, and the crocodile lizards did not recover after 1 week of treatment.

The bacterial community diversity was also significantly changed. The comparison of the Shannon index among the groups showed that the alpha diversity of the bacterial community in the ulcerated skin was significantly lower than those in healthy skin and environmental water and soil (Figure 4A). For the beta diversity, PCoA analysis and UPGMA clustering showed that the four groups were clustered differently (Figures 4B,C). PERMANOVA test also supported this clustering with *P* < 0.05 (Figure 4B). These results were well-confirmed by the samples collected from Gandong station in 2020 (Supplementary Figure 7). The lower community diversity indicates that there were some bacteria enriched in the ulcerated skin. Indeed, 9 bacterial OTUs were significantly enriched in the ulcerated skin, which accounted for 60.44% of the total bacteria (Supplementary Figure 3). In addition, the 9 enriched OTUs were shared in all ulcerated skin samples.

**Figure 5:**
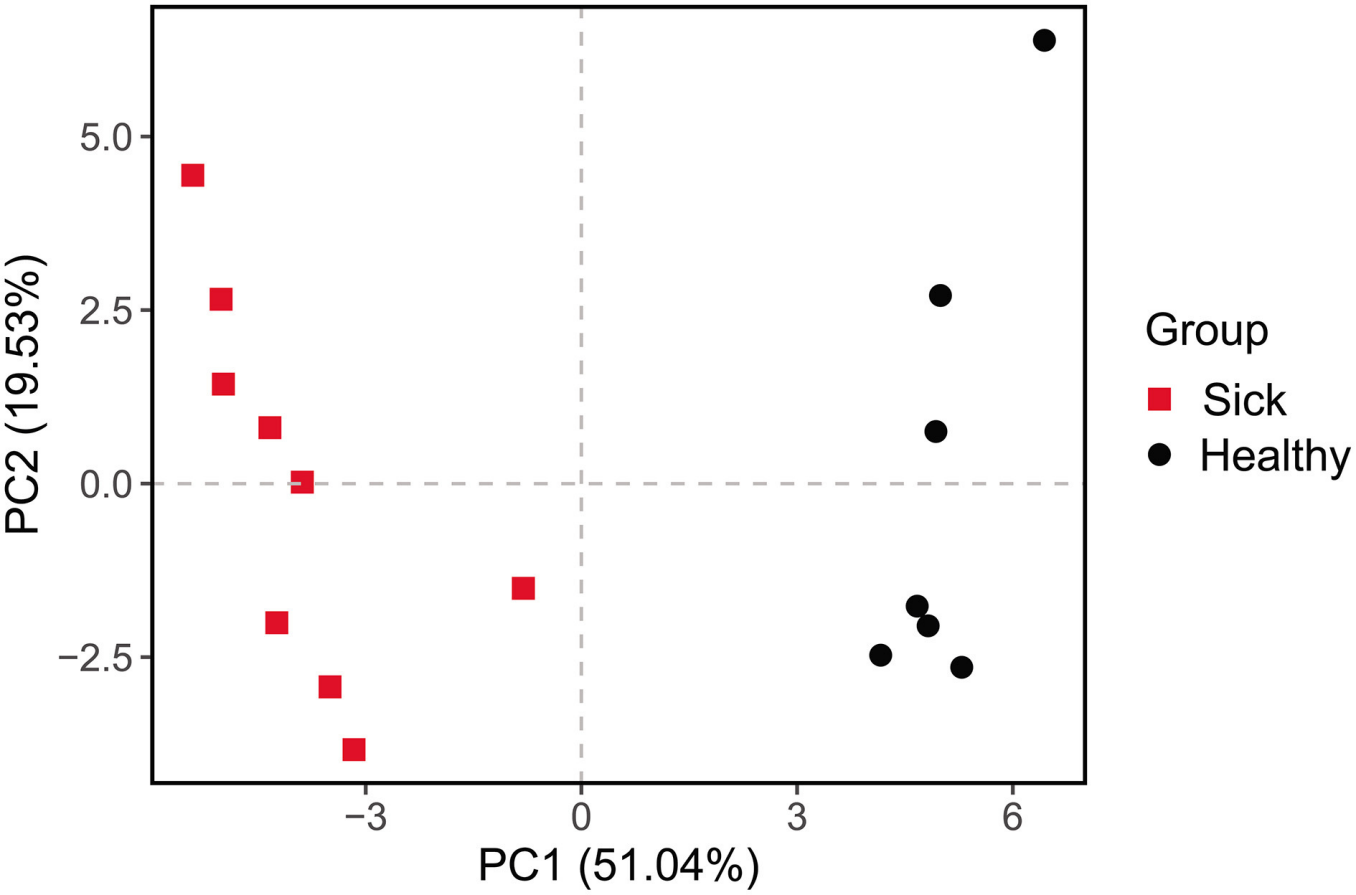
Principal components analysis (PCA) of function of the crocodile lizard cutaneous bacteria. Numbers inside the parenthesis in the axis label show the percentage variation explained by each PC. Function was predicted at level 2 of the KEGG pathway.

The differences between the ulcerated skin and the healthy skin were also reflected in the predicted function of the skin bacterial microbiome. Based on the KEGG database, the functions of the skin bacteria in crocodile lizards included metabolism, genetic information processing, environmental information processing, cellular processes, and disease at the level 1 (Supplementary Figure 4). However, the function of the ulcerated skin and healthy skin separated at level 2 (Figure 5), which indicated that the function of the bacterial community in ulcerated skin has significantly changed.

### Skin Ulcer Altered the Fungal Community Structure of the Skin

**Figure 6:**
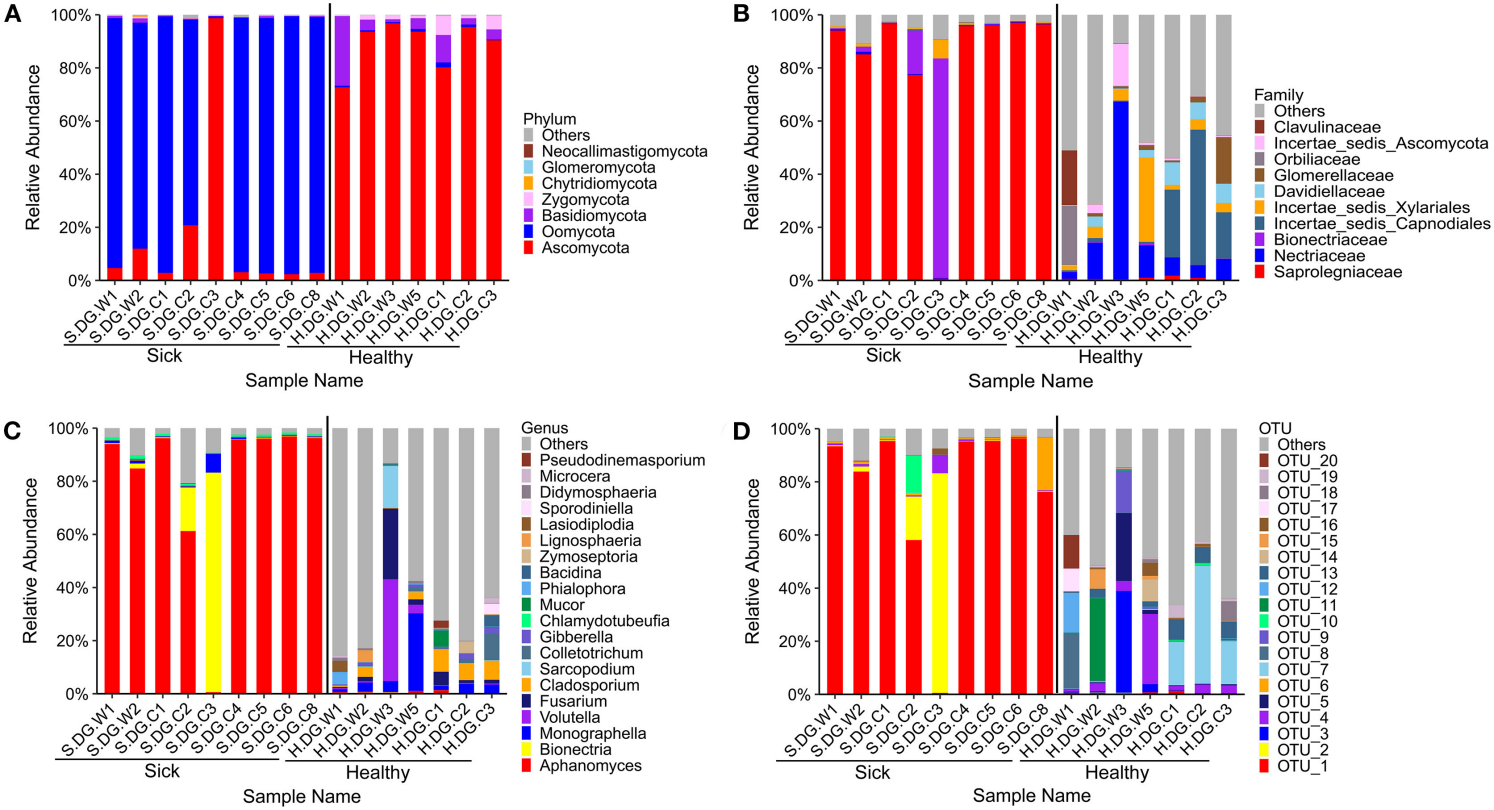
Composition of cutaneous fungi of crocodile lizard at the phylum **(A)**, family **(B)**, genus **(C)**, and OTU **(D)** levels. The taxa with abundance of top 7, 10, 20, 20 were showed at the phylum, family, genus and OTU levels. The rest taxa with low abundance were included in “Others.” Group boundaries were separated by black vertical lines.

The composition of fungal community in the ulcerated skin was obviously different with that in healthy skin. Only a few fungal species were found in the ulcerated skin. At the OTU level, it was dominated by OTU_1 *Aphanomyces sinensis* (average: 77.19%, maximum: 96.40%) (Figure 6D). This was followed by OTU_2 *Bionectria ochroleuca*. However, the distribution of OTU_2 was uneven among the samples. It was only highly abundant in samples S.DG.C2 (16.38%) and S.DG.C3 (82.65%) (Figure 6D). The sequencing results from Gandong station in 2020 confirmed a change in fungal composition in the ulcerated skin. However, the experiment in 2020 did not detect the high abundance of *Aphanomyce*, with *Cladosporium* and *Fusarium* as the dominant fungi (Supplementary Figure 8).

**Figure 7:**
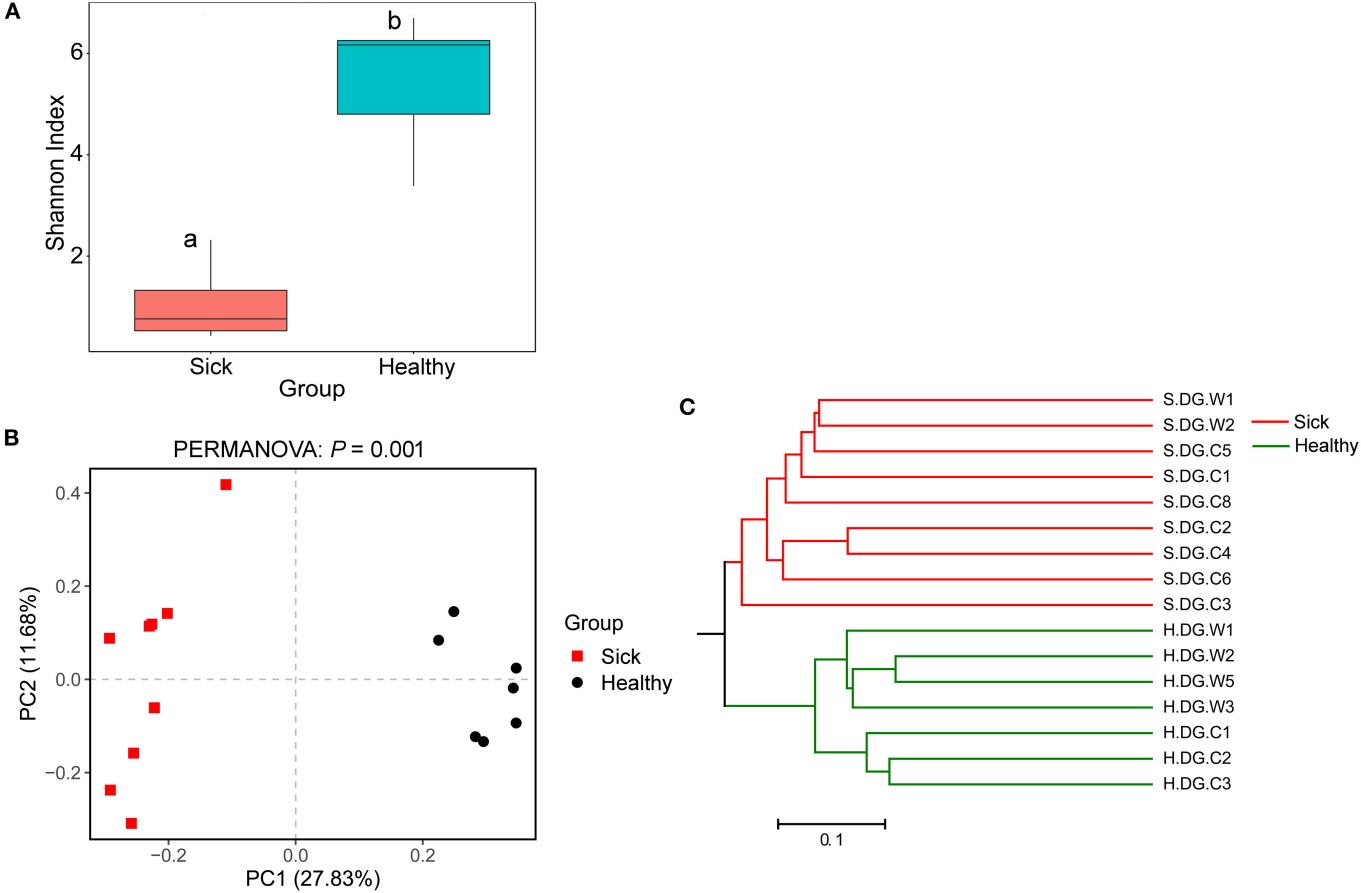
Community diversity of the cutaneous mycobiome of crocodile lizards. **(A)** Alpha diversity indicated by Shannon index. Data with different superscript letters are significantly different (*P* < 0.05) according to Wilcoxon test. **(B)** Beta diversity indicated by principal coordinate analysis (PCoA) and based on the unweighted UniFrac distance matrix. Numbers inside the parenthesis in the axis label show the percentage variation explained by each PC. *P*-value of PERMANOVA test is noted at the top of PCoA plot. **(C)** Beta diversity indicated by the UPGMA cluster based on the unweighted UniFrac distance matrix.

Regarding community diversity, the comparison of Shannon index showed that the alpha diversity in ulcerated skin was significantly lower than that in healthy skin (Figure 7A). For the beta diversity, the results of PCoA, UPGMA clustering, and PERMANOVA analysis were consistent, showing significant differences in the diversity of the fungal communities between ulcer and healthy skin (Figures 7B,C). The results also showed good reproducibility in the samples collected from Gandong station in 2020 (Supplementary Figure 9). The lower community diversity indicates that some fungi were enriched in the ulcerated skin. Three OTUs were significantly enriched in the ulcerated skin, namely OTU_1 *A. sinensis*, OTU_2 *B. ochroleuca*, and OTU_6 *Aphanomyces* sp., and these were shared in all ulcerated skin samples (Supplementary Figure 5). The total abundance of these three OTUs was 91.29%.

## Discussion

Our field investigation showed that skin ulcer in crocodile lizards has spread to the wild population and requires more attention owing to its high prevalence. The prevalence in the wild population seems related to the environmental ecology. In other words, the environmental ecology of the Dachai Chong stream with the lowest prevalence was best protected, whereas that of the Yusan Chong stream with the highest prevalence was the worst due to increased human disturbance. We observed that skin ulcer was severe in summer but mild in winter. This may be attributed to the increased replication of pathogens due to higher temperature, although this is difficult to verify in the wild population because crocodile lizards are difficult to locate in the winter when they are in hibernation. We speculated that humid and hot environments were more prone to disease outbreaks. Indeed, positive correlation between skin ulcer prevalence and the daily mean temperature were found but no relationship between prevalence of skin ulcer and the daily mean air humidity. This indicated that it is necessary to control the environmental temperature in summer to reduce skin diseases in crocodile lizard protection. In the first survey, the skin ulcer prevalence of subadults was significantly higher than that of adults, but the significance changed over time. This may be due to the fact that some subadults were assigned into the adult group in the later surveys as they grew up but were still sick, as well as the intervention of nature reserve staff in some individuals with this disease.

Furthermore, our findings provide information on the interaction of crocodile lizards with their environment. The skin microbiota is not only associated with diseases but also influenced by host species and environment. In non-human mammals, the host taxonomic order was the most significant factor influencing the skin microbiota, followed by the geographic location of the habitat (32). Bacterial, fungal, and viral communities on the human skin were largely stable over time despite the skin's exposure to the external environment (33). However, a study on Komodo dragons (*Varanus komodoensis*) revealed that the captive dragons and their enclosure had similar microbial community composition and species richness (34). This is inconsistent with our findings showing the skin microbiota of crocodile lizards was significantly different from their environmental water and soil microbial communities. However, the dynamic changes of healthy skin microbiota in crocodile lizards from Beilou and Gandong stations indicated that the environment had an impact on the skin microorganisms.

Microbial communities of healthy individuals are typically more diverse than those of the diseased. In dogs with allergies and atopic dermatitis, the skin bacterial community richness and diversity decreased (35, 36). Although no changes in bacterial community diversity were observed, the abundance of *Staphylococcus* was increased in allergic cats (37). Further, allergic skin diseases reduced the fungal community diversity in the different body sites of cats and dogs (8). The skin microbes in salamanders can inhibit the deadly chytrid fungus, but their protective effect was significantly reduced upon a decrease in their diversity (38, 39). There are only a few reports on the reptilian skin microbiota. In an endangered rattlesnake *Sistrurus catenatus*, snake fungal disease (SFD) altered both the skin bacterial and fungal diversity (16). Similarly, we observed alterations in the bacterial and fungal communities in crocodile lizards with skin ulcer. Both the bacterial and fungal diversity significantly decreased in ulcerated skin samples, and the dominant bacteria and fungi also changed, although the dominant bacteria and fungi were not completely consistent in two experiments. This phenomenon suggested that skin disease, such as skin ulcer in this study, was an important factor in altering the skin microbiota of crocodile lizards despite that the environment also played a role. In human, cutaneous microbiome was also altered in patient with pressure ulcer (40). These results are consistent with the idea that the microbiome in a healthy state is characterized by generalist symbionts, whereas that in a diseased state has “specialist” microorganisms that possess specific metabolic functions and an elevated virulence potential (2). In addition, certain opportunistic bacteria that live on the skin may in turn influence the course of the disease. Insights obtained indicate the need for exploring skin microbiota modulation as a potential therapeutic option for skin disease in wildlife conservation.

Unfortunately, this study cannot infer the primary pathogen directly using the sequencing results. According to the Koch's postulate, pathogens in the ulcerated skin of crocodile lizards must be present in every diseased sample. For the bacterial community, the 9 enriched OTUs in the ulcerated skin were shared in all lesions. Among these enriched OTUs, *Elizabethkingia miricola* was also the most dominant isolated strain. However, broad-spectrum antibiotics targeting the isolated dominant bacteria (minocycline and levofloxacin) did not play a significant role in treatment. This indicated that skin ulcer may not be primarily caused by bacteria. For the fungal community, the average abundance of *Aphanomyces* (OTU_1 and OTU_6) in ulcerated skin was as high as 80.20% and was shared in all ulcerated skin samples. *Aphanomyces* belongs to the family Saprolegniaceae and class Oomycetes, and are the pathogens of epizootic ulcerative syndrome, mainly characterized by ulcer and granuloma (41–46). They are listed as class II animal diseases in China and are harmful to fish, crustaceans, reptiles, and crops (41, 47–49). In reptiles, *Aphanomyces* has caused a mycosis outbreak in Chinese soft-shelled turtle in Japan in 2007–2009 (47). The pathogens *Aphanomyces* sp. NJM 0703 and *A. sinensis* NJM 0719 isolated from a Chinese soft-shelled turtle had 99.5% and 97% similarity, respectively, to our OTU_1. We had speculated that *Aphanomyces* were related to skin ulcer in crocodile lizards, especially *A. sinensis*, whose average abundance was 77.19% and can go as high as 96.40%. However, we did not detect high abundance of *Aphanomyces* in the sick crocodile lizards collected from Gandong station in 2020. Therefore, further studies on pathogen isolation and validation are required in future.

A better understanding of the skin microbiota provides insights into the mechanisms of pathogen emergence, fluctuations in health status of the host, and the modulation in therapeutic intervention to reduce disease impact. To the best of our knowledge, this is the first study on the relationship between skin microbiome and dermatosis in lizards, thus providing an increased understanding of the role of the skin microbiota on skin health and diseases. Because skin diseases cause widespread morbidity in crocodile lizards, understanding the skin microbiome will provide opportunities to create effective conservation management programs.

## Conclusion

It should be noticed that skin ulcer has been prevalent in both captive and the wild populations of crocodile lizards, and care should be taken to cool them in summer to reduce the occurrence of skin disease. The skin microbiota of crocodile lizards was different from the microbial communities of the environment. Skin ulcer is significantly related to changes in the skin microbiota despite the impact of environment. Therefore, we should also pay attention to the modulation of skin microbiota in wildlife conservation.

These fundamental information on wild and captive crocodile lizards provide a reference for the conservation of this endangered reptile. Future studies should include samples across spatial and temporal variability to yield more accurate skin microbial community baseline.

## Data Availability Statement

All raw sequences obtained in this study have been deposited in the Sequence Read Archive (SRA) database in NCBI under accession numbers SRP187407 and SRP187549, the BioProject numbers were PRJNA525317 and PRJNA525561.

## Ethics Statement

The animal study was reviewed and approved by the Committee on the Ethics of Animal Experiments of the Institute of Zoology, Guangdong Academy of Sciences. Written informed consent was obtained from the owners for the participation of their animals in this study.

## Author Contributions

JC: conceptualization. HJ, LL, and XZ: formal analysis. HJ, SL, JZ, WH, and JH: investigation. HJ: writing—original draft. HJ and JC: writing—review and editing. All authors contributed to the article and approved the submitted version.

## Funding

This project was supported by the Guangzhou Science and Technology Project (202102020343) and project of National Forestry and Grassland Administration (2020076079).

## Conflict of Interest

The authors declare that the research was conducted in the absence of any commercial or financial relationships that could be construed as a potential conflict of interest.

## Publisher's Note

All claims expressed in this article are solely those of the authors and do not necessarily represent those of their affiliated organizations, or those of the publisher, the editors and the reviewers. Any product that may be evaluated in this article, or claim that may be made by its manufacturer, is not guaranteed or endorsed by the publisher.
