# Supplementary material for: Skin Microbiota Was Altered in Crocodile Lizards (Shinisaurus crocodilurus) With Skin Ulcer
Source: Front Vet Sci. 2022 Feb 14;9:817490. doi: 10.3389/fvets.2022.817490 (PMC8884271; doi:10.3389/fvets.2022.817490)
Supplement: Supplementary file 1 [file Data_Sheet_1.DOCX]

**Supporting Information 1**

**Table S1** Statistical test results of the differences in prevalence between female and male crocodile lizards using Pearson's chi-squared test or Fisher's exact test.

| Survey Date |  | Number of Cases | | |  | Statistical Test Result |
| --- | --- | --- | --- | --- | --- | --- |
|  | Gender | Sick | Healthy | Total | Prevalence |  |
| 20170628-29 | Female | 18 | 63 | 81 | 22.22% | χ^2^ = 0.4812, df = 1, *P* = 0.4879 |
|  | Male | 12 | 56 | 68 | 17.65% |  |
|  |  |  |  |  |  |  |
| 20170704-05 | Female | 46 | 34 | 80 | 57.50% | χ^2^ = 0.9974, df = 1, *P* = 0.3179 |
|  | Male | 33 | 34 | 67 | 49.25% |  |
|  |  |  |  |  |  |  |
| 20170712-13 | Female | 12 | 68 | 80 | 15.00% | χ^2^ = 2.0255, df = 1, *P* = 0.1547 |
|  | Male | 5 | 62 | 67 | 7.46% |  |
|  |  |  |  |  |  |  |
| 20170718-20 | Female | 28 | 52 | 80 | 35.00% | χ^2^ = 1.5909, df = 1, *P* = 0.2072 |
|  | Male | 17 | 50 | 67 | 25.37% |  |
|  |  |  |  |  |  |  |
| 20170801-02 | Female | 18 | 60 | 78 | 23.08% | χ^2^ = 1.4336, df = 1, *P* = 0.2312 |
|  | Male | 10 | 56 | 66 | 15.15% |  |
|  |  |  |  |  |  |  |
| 20170815-17 | Female | 11 | 67 | 78 | 14.10% | χ^2^ = 0.1816, df = 1, *P* = 0.6700 |
|  | Male | 11 | 55 | 66 | 16.67% |  |
|  |  |  |  |  |  |  |
| 20170821-22 | Female | 5 | 73 | 78 | 6.41% | *P* = 0.4533 (Fisher's exact test) |
|  | Male | 2 | 64 | 66 | 3.03% |  |
|  |  |  |  |  |  |  |
| 20170829-31 | Female | 13 | 64 | 77 | 16.88% | χ^2^ = 0.0023, df = 1, *P* = 0.9618 |
|  | Male | 11 | 53 | 64 | 17.19% |  |
|  |  |  |  |  |  |  |
| 20170905-06 | Female | 7 | 69 | 76 | 9.21% | *P* = 0.3481 (Fisher's exact test) |
|  | Male | 3 | 60 | 63 | 4.76% |  |
|  |  |  |  |  |  |  |
| 20170912 | Female | 3 | 72 | 75 | 4.00% | *P* = 0.7011 (Fisher's exact test) |
|  | Male | 4 | 58 | 62 | 6.45% |  |
|  |  |  |  |  |  |  |
| 20170927 | Female | 4 | 70 | 74 | 5.41% | *P* = 0.3775 (Fisher's exact test) |
|  | Male | 1 | 60 | 61 | 1.64% |  |
|  |  |  |  |  |  |  |
| 20171010 | Female | 0 | 74 | 74 | 0 | *P* = 0.4519 (Fisher's exact test) |
|  | Male | 1 | 60 | 61 | 1.64% |  |
|  |  |  |  |  |  |  |
| 20171018 | Female | 0 | 74 | 74 | 0 | *P* = 0.4519 (Fisher's exact test) |
|  | Male | 1 | 60 | 61 | 1.64% |  |
|  |  |  |  |  |  |  |
| 20180425-27 | Female | 2 | 91 | 93 | 2.15% | *P* = 0.5069 (Fisher's exact test) |
|  | Male | 0 | 70 | 70 | 0 |  |
|  |  |  |  |  |  |  |
| 20180508-09 | Female | 0 | 92 | 92 | 0 | *P* = 0.4321 (Fisher's exact test) |
|  | Male | 1 | 69 | 70 | 1.43% |  |
|  |  |  |  |  |  |  |
| 20180515 | Female | 2 | 90 | 92 | 2.17% | *P* = 1.0000 (Fisher's exact test) |
|  | Male | 1 | 69 | 70 | 1.43% |  |
|  |  |  |  |  |  |  |
| 20180520 | Female | 2 | 90 | 92 | 2.17% | *P* = 0.5062 (Fisher's exact test) |
|  | Male | 0 | 70 | 70 | 0 |  |
|  |  |  |  |  |  |  |
| 20180529 | Female | 2 | 90 | 92 | 2.17% | *P* = 0.5062 (Fisher's exact test) |
|  | Male | 0 | 70 | 70 | 0 |  |
|  |  |  |  |  |  |  |
| 20180607 | Female | 2 | 89 | 91 | 2.20% | *P* = 0.5064 (Fisher's exact test) |
|  | Male | 0 | 69 | 69 | 0 |  |
|  |  |  |  |  |  |  |
| 20180620-21 | Female | 2 | 89 | 91 | 2.20% | *P* = 1.0000 (Fisher's exact test) |
|  | Male | 1 | 67 | 68 | 1.47% |  |
|  |  |  |  |  |  |  |
| 20180627 | Female | 2 | 89 | 91 | 2.20% | *P* = 1.0000 (Fisher's exact test) |
|  | Male | 1 | 67 | 68 | 1.47% |  |
|  |  |  |  |  |  |  |
| 20180710-12 | Female | 4 | 87 | 91 | 4.40% | *P* = 0.3277 (Fisher's exact test) |
|  | Male | 6 | 62 | 68 | 8.82% |  |
|  |  |  |  |  |  |  |
| 20180717 | Female | 3 | 88 | 91 | 3.30% | *P* = 1.0000 (Fisher's exact test) |
|  | Male | 2 | 66 | 68 | 2.94% |  |
|  |  |  |  |  |  |  |
| 20180814-15 | Female | 1 | 90 | 91 | 1.10% | *P* = 0.0846 (Fisher's exact test) |
|  | Male | 5 | 63 | 68 | 7.35% |  |
|  |  |  |  |  |  |  |
| 20180831 | Female | 1 | 90 | 91 | 1.10% | *P* = 1.0000 (Fisher's exact test) |
|  | Male | 0 | 68 | 68 | 0 |  |
|  |  |  |  |  |  |  |
| 20180912 | Female | 1 | 90 | 91 | 1.10% | *P* = 1.0000 (Fisher's exact test) |
|  | Male | 0 | 68 | 68 | 0 |  |
|  |  |  |  |  |  |  |
| 20180919 | Female | 4 | 87 | 91 | 4.40% | *P* = 1.0000 (Fisher's exact test) |
|  | Male | 3 | 65 | 68 | 4.41% |  |
|  |  |  |  |  |  |  |
| 20181009 | Female | 1 | 90 | 91 | 1.10% | *P* = 1.0000 (Fisher's exact test) |
|  | Male | 0 | 68 | 68 | 0 |  |
|  |  |  |  |  |  |  |
| 20181024 | Female | 0 | 63 | 63 | 0 | *P* = 1.0000 (Fisher's exact test) |
|  | Male | 0 | 50 | 50 | 0 |  |
|  |  |  |  |  |  |  |
| 20190502 | Female | 13 | 77 | 90 | 14.44% | *P* = 0.0598 (Fisher's exact test) |
|  | Male | 3 | 66 | 69 | 4.35% |  |
|  |  |  |  |  |  |  |
| 20190613 | Female | 12 | 65 | 77 | 15.58% | χ^2^ = 0.2480, df = 1, *P* = 0.6185 |
|  | Male | 12 | 52 | 64 | 18.75% |  |
|  |  |  |  |  |  |  |
| 20190619-20 | Female | 17 | 59 | 76 | 22.37% | χ^2^ = 0.2301, df = 1, *P* = 0.6315 |
|  | Male | 12 | 51 | 63 | 19.05% |  |
|  |  |  |  |  |  |  |
| 20190625-26 | Female | 11 | 65 | 76 | 14.47% | χ^2^ = 0.3455, df = 1, *P* = 0.5567 |
|  | Male | 7 | 56 | 63 | 11.11% |  |
|  |  |  |  |  |  |  |
| 20190709 | Female | 21 | 54 | 75 | 28.00% | χ^2^ = 0.6038, df = 1, *P* = 0.4371 |
|  | Male | 14 | 49 | 63 | 22.22% |  |
|  |  |  |  |  |  |  |
| 20190717-18 | Female | 20 | 54 | 74 | 27.03% | χ^2^ = 0.6453, df = 1, *P* = 0.4218 |
|  | Male | 21 | 42 | 63 | 33.33% |  |
|  |  |  |  |  |  |  |
| 20190731-0801 | Female | 9 | 65 | 74 | 12.16% | χ^2^ = 0.0090, df = 1, *P* = 0.9244 |
|  | Male | 8 | 55 | 63 | 12.70% |  |
|  |  |  |  |  |  |  |
| 20190911 | Female | 5 | 17 | 22 | 22.73% | *P* = 0.7076 (Fisher's exact test) |
|  | Male | 5 | 10 | 15 | 33.33% |  |
|  |  |  |  |  |  |  |
| 20190916 | Female | 2 | 20 | 22 | 9.09% | *P* = 0.0842 (Fisher's exact test) |
|  | Male | 5 | 9 | 14 | 35.71% |  |
|  |  |  |  |  |  |  |
| 20190925 | Female | 2 | 20 | 22 | 9.09% | *P* = 0.3566 (Fisher's exact test) |
|  | Male | 3 | 11 | 14 | 21.43% |  |
|  |  |  |  |  |  |  |
| 20191012 | Female | 0 | 22 | 22 | 0 | *P* = 0.3889 (Fisher's exact test) |
|  | Male | 1 | 13 | 14 | 7.14% |  |

**Table S2** Statistical test results of the differences in prevalence between adult and sub-adult crocodile lizards using Pearson's chi-squared test or Fisher's exact test.

| Survey Date |  | Number of Cases | | |  | Statistical Test Result |
| --- | --- | --- | --- | --- | --- | --- |
|  | Age | Sick | Healthy | Total | Prevalence |  |
| **20170628-29*** | Adult | 3 | 94 | 97 | 3.09% | **χ^2^ = 50.1960, df = 1, *P* = 1.391e-12** |
|  | Sub-adult | 27 | 25 | 52 | 51.92% |  |
|  |  |  |  |  |  |  |
| 20170704-05 | Adult | 57 | 39 | 96 | 59.38% | χ^2^ = 3.5324, df = 1, *P* = 0.0602 |
|  | Sub-adult | 22 | 29 | 51 | 43.14% |  |
|  |  |  |  |  |  |  |
| **20170712-13*** | Adult | 1 | 95 | 96 | 1.04% | **χ^2^ = 29.9600, df = 1, *P* = 4.411e-08** |
|  | Sub-adult | 16 | 35 | 51 | 31.37% |  |
|  |  |  |  |  |  |  |
| 20170718-20 | Adult | 30 | 66 | 96 | 31.25% | χ^2^ = 0.0530, df = 1, *P* = 0.8179 |
|  | Sub-adult | 15 | 36 | 51 | 29.41% |  |
|  |  |  |  |  |  |  |
| 20170801-02 | Adult | 18 | 76 | 94 | 19.15% | χ^2^ = 0.0151, df = 1, *P* = 0.9022 |
|  | Sub-adult | 10 | 40 | 50 | 20.00% |  |
|  |  |  |  |  |  |  |
| 20170815-17 | Adult | 17 | 77 | 94 | 18.09% | χ^2^ = 1.6483, df = 1, *P* = 0.1992 |
|  | Sub-adult | 5 | 45 | 50 | 10.00% |  |
|  |  |  |  |  |  |  |
| 20170821-22 | Adult | 3 | 91 | 94 | 3.19% | *P* = 0.2365 (Fisher's exact test) |
|  | Sub-adult | 4 | 46 | 50 | 8.00% |  |
|  |  |  |  |  |  |  |
| 20170829-31 | Adult | 19 | 72 | 91 | 20.88% | χ^2^ = 2.7041, df = 1, *P* = 0.1001 |
|  | Sub-adult | 5 | 45 | 50 | 10.00% |  |
|  |  |  |  |  |  |  |
| 20170905-06 | Adult | 8 | 82 | 90 | 8.89% | *P* = 0.1595 (Fisher's exact test) |
|  | Sub-adult | 1 | 48 | 49 | 2.04% |  |
|  |  |  |  |  |  |  |
| **20170912*** | Adult | 7 | 81 | 88 | 7.95% | ***P* = 0.0498 (Fisher's exact test)** |
|  | Sub-adult | 0 | 49 | 49 | 0 |  |
|  |  |  |  |  |  |  |
| 20170927 | Adult | 1 | 85 | 86 | 1.16% | *P* = 0.0581 (Fisher's exact test) |
|  | Sub-adult | 4 | 45 | 49 | 8.16% |  |
|  |  |  |  |  |  |  |
| 20171010 | Adult | 1 | 85 | 86 | 1.16% | *P* = 1.0000 (Fisher's exact test) |
|  | Sub-adult | 0 | 49 | 49 | 0 |  |
|  |  |  |  |  |  |  |
| 20171018 | Adult | 1 | 85 | 86 | 1.16% | *P* = 1.0000 (Fisher's exact test) |
|  | Sub-adult | 0 | 49 | 49 | 0 |  |
|  |  |  |  |  |  |  |
| 20180425-27 | Adult | 2 | 129 | 131 | 1.53% | *P* = 1.0000 (Fisher's exact test) |
|  | Sub-adult | 0 | 32 | 32 | 0 |  |
|  |  |  |  |  |  |  |
| 20180508-09 | Adult | 5 | 126 | 131 | 3.82% | *P* = 0.5843 (Fisher's exact test) |
|  | Sub-adult | 0 | 31 | 31 | 0 |  |
|  |  |  |  |  |  |  |
| 20180515 | Adult | 3 | 128 | 131 | 2.29% | *P* = 1.0000 (Fisher's exact test) |
|  | Sub-adult | 0 | 31 | 31 | 0 |  |
|  |  |  |  |  |  |  |
| 20180520 | Adult | 2 | 129 | 131 | 1.53% | *P* = 1.0000 (Fisher's exact test) |
|  | Sub-adult | 0 | 31 | 31 | 0 |  |
|  |  |  |  |  |  |  |
| 20180529 | Adult | 2 | 129 | 131 | 1.53% | *P* = 1.0000 (Fisher's exact test) |
|  | Sub-adult | 0 | 31 | 31 | 0 |  |
|  |  |  |  |  |  |  |
| 20180607 | Adult | 2 | 127 | 129 | 1.55% | *P* = 1.0000 (Fisher's exact test) |
|  | Sub-adult | 0 | 31 | 31 | 0 |  |
|  |  |  |  |  |  |  |
| 20180620-21 | Adult | 3 | 125 | 128 | 2.34% | *P* = 1.0000 (Fisher's exact test) |
|  | Sub-adult | 0 | 31 | 31 | 0 |  |
|  |  |  |  |  |  |  |
| 20180627 | Adult | 3 | 125 | 128 | 2.34% | *P* = 1.0000 (Fisher's exact test) |
|  | Sub-adult | 0 | 31 | 31 | 0 |  |
|  |  |  |  |  |  |  |
| 20180710-12 | Adult | 8 | 120 | 128 | 6.25% | *P* = 1.0000 (Fisher's exact test) |
|  | Sub-adult | 2 | 29 | 31 | 6.45% |  |
|  |  |  |  |  |  |  |
| 20180717 | Adult | 5 | 123 | 128 | 3.91% | *P* = 0.5839 (Fisher's exact test) |
|  | Sub-adult | 0 | 31 | 31 | 0 |  |
|  |  |  |  |  |  |  |
| 20180814-15 | Adult | 6 | 121 | 127 | 4.72% | *P* = 0.5985 (Fisher's exact test) |
|  | Sub-adult | 0 | 31 | 31 | 0 |  |
|  |  |  |  |  |  |  |
| 20180831 | Adult | 1 | 126 | 127 | 0.79% | *P* = 1.0000 (Fisher's exact test) |
|  | Sub-adult | 0 | 31 | 31 | 0 |  |
|  |  |  |  |  |  |  |
| 20180912 | Adult | 1 | 126 | 127 | 0.79% | *P* = 1.0000 (Fisher's exact test) |
|  | Sub-adult | 0 | 31 | 31 | 0 |  |
|  |  |  |  |  |  |  |
| 20180919 | Adult | 6 | 121 | 127 | 4.72% | *P* = 1.0000 (Fisher's exact test) |
|  | Sub-adult | 1 | 30 | 31 | 3.23% |  |
|  |  |  |  |  |  |  |
| 20181009 | Adult | 1 | 126 | 127 | 0.79% | *P* = 1.0000 (Fisher's exact test) |
|  | Sub-adult | 0 | 31 | 31 | 0 |  |
|  |  |  |  |  |  |  |
| 20181024 | Adult | 0 | 95 | 95 | 0 | *P* = 1.0000 (Fisher's exact test) |
|  | Sub-adult | 0 | 18 | 18 | 0 |  |
|  |  |  |  |  |  |  |
| **20190502*** | Adult | 0 | 107 | 107 | 0 | **χ^2^ = 36.6070, df = 1, *P* = 1.445e-09** |
|  | Sub-adult | 16 | 36 | 52 | 30.77% |  |
|  |  |  |  |  |  |  |
| 20190613 | Adult | 14 | 85 | 99 | 14.14% | χ^2^ = 1.9516, df = 1, *P* = 0.1624 |
|  | Sub-adult | 10 | 32 | 42 | 23.81% |  |
|  |  |  |  |  |  |  |
| 20190619-20 | Adult | 21 | 76 | 97 | 21.65% | χ^2^ = 0.1202, df = 1, *P* = 0.7288 |
|  | Sub-adult | 8 | 34 | 42 | 19.05% |  |
|  |  |  |  |  |  |  |
| 20190625-26 | Adult | 9 | 88 | 97 | 9.28% | χ^2^ = 3.8384, df = 1, *P* = 0.0501 |
|  | Sub-adult | 9 | 33 | 42 | 21.43% |  |
|  |  |  |  |  |  |  |
| **20190709*** | Adult | 15 | 82 | 97 | 15.46% | **χ^2^ = 16.8990, df = 1, *P* = 3.943e-05** |
|  | Sub-adult | 20 | 21 | 41 | 48.78% |  |
|  |  |  |  |  |  |  |
| **20190717-18*** | Adult | 22 | 75 | 97 | 22.68% | **χ^2^ = 8.3193, df = 1, *P* = 0.0039** |
|  | Sub-adult | 19 | 21 | 40 | 47.50% |  |
|  |  |  |  |  |  |  |
| 20190731-0801 | Adult | 12 | 85 | 97 | 12.37% | *P* = 1.0000 (Fisher's exact test) |
|  | Sub-adult | 5 | 35 | 40 | 12.50% |  |
|  |  |  |  |  |  |  |
| 20190911 | Adult | 8 | 24 | 32 | 25.00% | *P* = 0.5974 (Fisher's exact test) |
|  | Sub-adult | 2 | 3 | 5 | 40.00% |  |
|  |  |  |  |  |  |  |
| 20190916 | Adult | 7 | 24 | 31 | 22.58% | *P* = 0.5590 (Fisher's exact test) |
|  | Sub-adult | 0 | 5 | 5 | 0 |  |
|  |  |  |  |  |  |  |
| 20190925 | Adult | 5 | 26 | 31 | 16.13% | *P* = 1.0000 (Fisher's exact test) |
|  | Sub-adult | 0 | 5 | 5 | 0 |  |
|  |  |  |  |  |  |  |
| 20191012 | Adult | 1 | 30 | 31 | 3.23% | *P* = 1.0000 (Fisher's exact test) |
|  | Sub-adult | 0 | 5 | 5 | 0 |  |

*, *P* < 0.05.

**Table S3** Sensitivities of the bacteria isolated from the ulcerated skin of crocodile lizards (*Shinisaurus crocodilurus*) to various antibiotics

| Strain | Minocycline | | Levofloxacin | | Ampicillin | | Rifampicin | | Kanamycin | | Gentamicin | |
| --- | --- | --- | --- | --- | --- | --- | --- | --- | --- | --- | --- | --- |
|  | ZD (mm) | IC | ZD (mm) | IC | ZD (mm) | IC | ZD (mm) | IC | ZD (mm) | IC | ZD (mm) | IC |
| *Elizabethkingia miricola* D31-15 | 18.3 | S | 21.2 | S | — | R | 15.2 | R | — | R | — | R |
| *Citrobacter* sp. D31-44 | 12.5 | I | 19.1 | S | — | R | — | R | 15.0 | I | 14.0 | I |
| *Enterobacter bugandensis* D31-17 | 15.1 | I | 25.3 | S | — | R | — | R | 16.0 | I | 17.0 | S |
| *Enterobacter hormaechei* D31-72 | 12.3 | I | 28.0 | S | — | R | — | R | 18.5 | S | 17.3 | S |
| *Elizabethkingia ursingii* D31-2 | 19.1 | S | 25.0 | S | — | R | — | R | — | R | — | R |
| *Providencia rettgeri* D31-47 | 10.0 | R | 11.8 | R | — | R | — | R | — | R | 9.5 | R |
| *Acinetobacter bereziniae* D31-1 | 21.8 | S | 16.0 | I | — | R | 8.0 | R | 21.0 | S | 17.5 | S |
| *Acinetobacter junii* D31-9 | 21.5 | S | 19.0 | S | 12.0 | R | 11.4 | R | 19.5 | S | 18.0 | S |
| *Stenotrophomonas maltophilia* D31-20 | 19.3 | S | 24.2 | S | — | R | 9.0 | R | — | R | — | R |
| *Bacillus megaterium* D31-12 | 19.0 | S | 19.8 | S | — | R | 18.8 | I | 20.5 | S | 18.0 | S |
| *Arthrobacter protophormiae* D31-52 | 23.5 | S | 17.3 | I | 24.5 | S | 25.2 | S | 15.5 | I | 16.2 | S |

ZD, inhibitory zone diameter; IC, interpretive categories; S, susceptible; I, intermediate; R, resistant.

**Table S3 (continued)** Sensitivities of the bacteria isolated from the ulcerated skin of crocodile lizards (*Shinisaurus crocodilurus*) to various antibiotics

| Strain | Streptomycin | | Cefoxitin | | Erythromycin | | | Clarithromycin | | Ciprofloxacin | | Piperacillin | |
| --- | --- | --- | --- | --- | --- | --- | --- | --- | --- | --- | --- | --- | --- |
|  | ZD (mm) | IC | ZD (mm) | IC | | ZD (mm) | IC | ZD (mm) | IC | ZD (mm) | IC | ZD (mm) | IC |
| *Elizabethkingia* *miricola* D31-15 | — | R | — | R | | 18.0 | I | 22.5 | S | 17.5 | I | 9.0 | R |
| *Citrobacter* sp. D31-44 | 10.5 | R | — | R | | — | R | 8.0 | R | 23.5 | S | 14.8 | R |
| *Enterobacter bugandensis* D31-17 | 13.0 | I | — | R | | 11.0 | R | 11.0 | R | 25.0 | S | 16.5 | R |
| *Enterobacter hormaechei* D31-72 | 13.5 | I | — | R | | — | R | 10.0 | R | 25.0 | S | 18.0 | I |
| *Elizabethkingia ursingii* D31-2 | — | R | — | R | | — | R | — | R | 18.5 | I | 10.0 | R |
| *Providencia rettgeri* D31-47 | — | R | — | R | | — | R | — | R | 8.0 | R | 8.0 | R |
| *Acinetobacter bereziniae* D31-1 | 15.5 | S | — | R | | 16.5 | I | 16.0 | I | — | R | 13.5 | R |
| *Acinetobacter junii* D31-9 | 17.3 | S | — | R | | 22.5 | I | 20.5 | S | 14.0 | R | 18.5 | I |
| *Stenotrophomonas maltophilia* D31-20 | — | R | — | R | | — | R | — | R | 20.5 | I | — | R |
| *Bacillus megaterium* D31-12 | 12.0 | I | 32.5 | S | | 25.0 | S | 24.5 | S | 11.2 | R | 17.5 | I |
| *Arthrobacter protophormiae* D31-52 | 16.5 | S | 26.0 | S | | 23.5 | S | 24.5 | S | 14.5 | R | 19.0 | I |

ZD, inhibitory zone diameter; IC, interpretive categories; S, susceptible; I, intermediate; R, resistant.

**
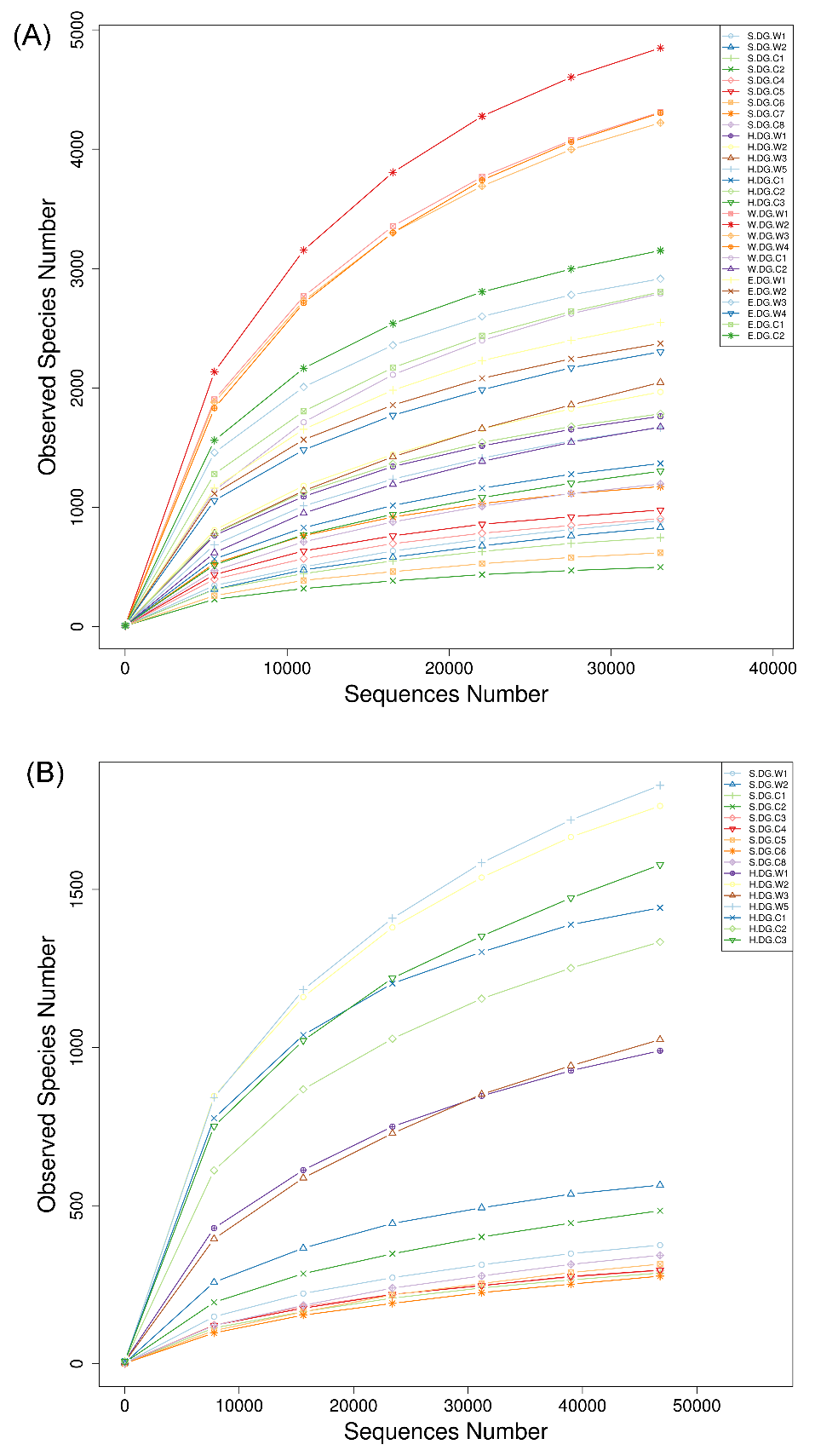
**

**Figure S1** Rarefaction curves of each sample in (A) 16S rDNA and (B) ITS1 sequencing.

**

**

**Figure S2** Relative abundances of bacteria isolated from the ulcerated skin of crocodile lizards.


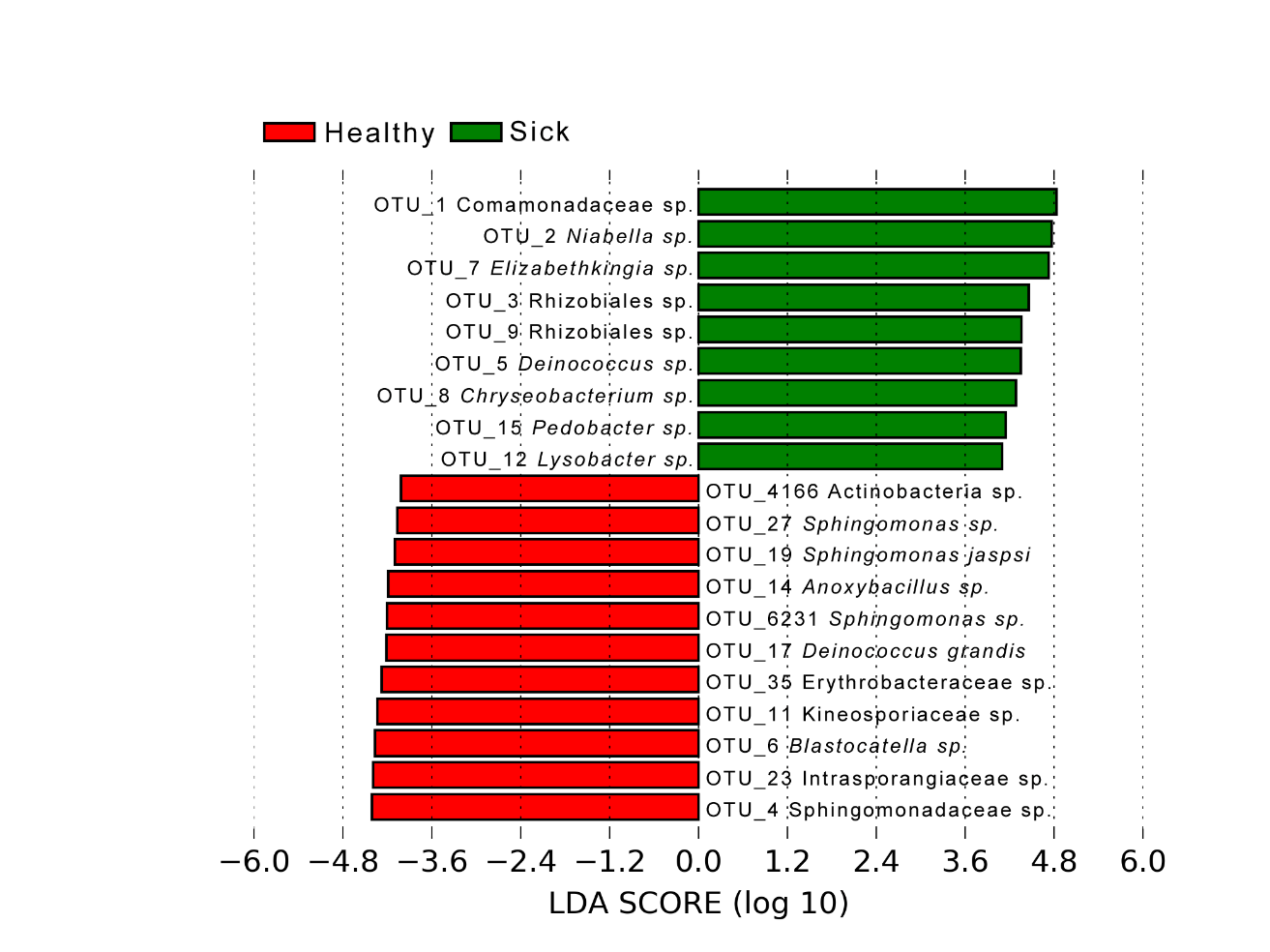


**Figure S3** Bacterial OTUs with significant abundance difference between the ulcerated and healthy skin samples. The highlighted taxa are significantly enriched in the group that corresponds to each color.

**
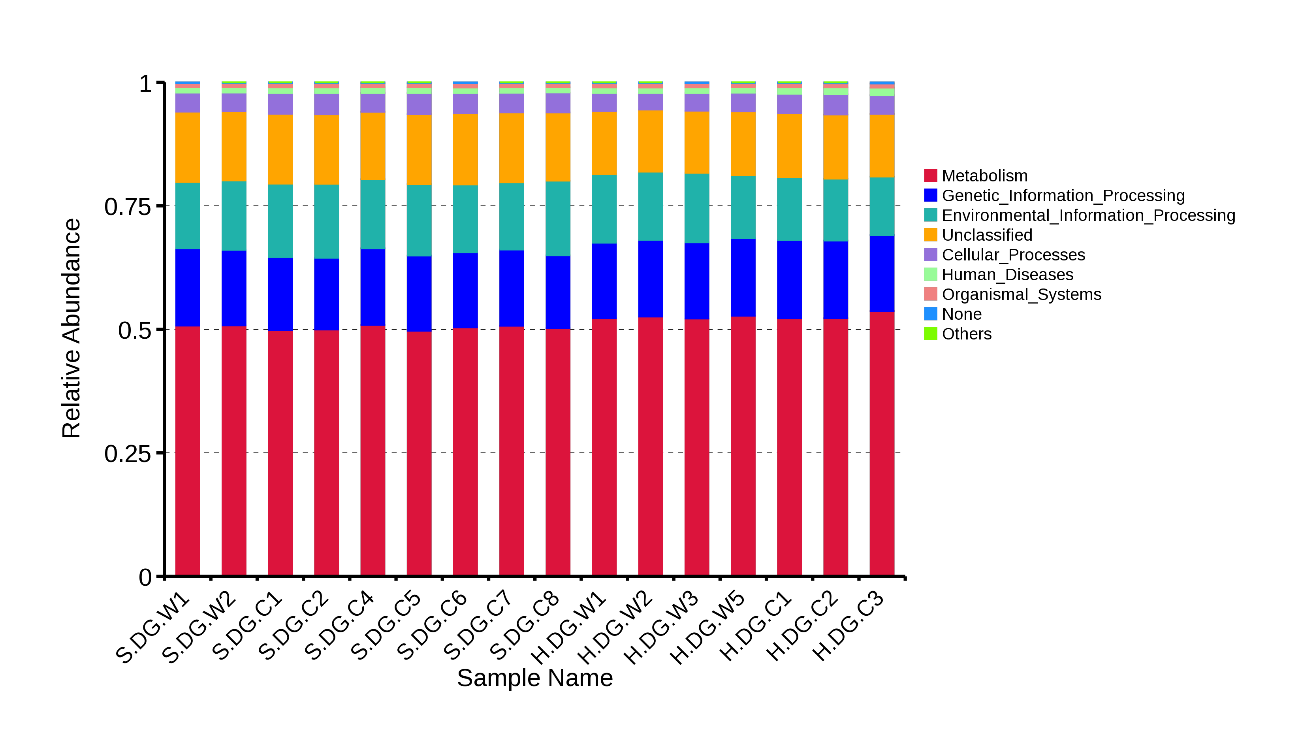
**

**Figure S4** Relative abundances of function of the crocodile lizard cutaneous bacteria. Function was predicted at level 1 of the KEGG pathway.


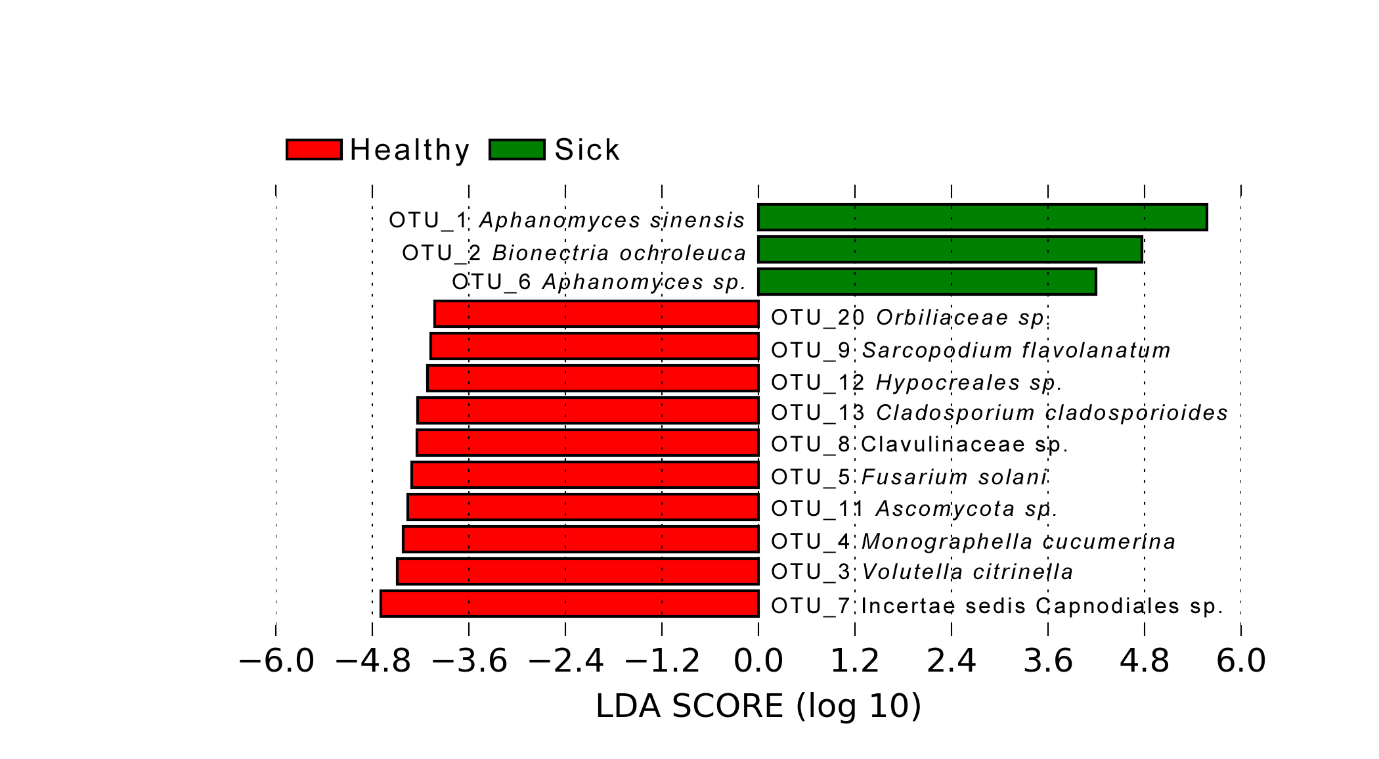


**Figure S5** Fungal OTUs with significant abundance difference between the ulcerated and healthy skin. The highlighted taxa are significantly enriched in the group that corresponds to each color.
