# Supplementary material for: Skin Microbiota Was Altered in Crocodile Lizards (Shinisaurus crocodilurus) With Skin Ulcer
Source: Front Vet Sci. 2022 Feb 14;9:817490. doi: 10.3389/fvets.2022.817490 (PMC8884271; doi:10.3389/fvets.2022.817490)
Supplement: Supplementary file 2 [file Data_Sheet_2.DOCX]

**Supporting Information 2** **Sequencing Results of The Samples Collected from Gandong Station In 2020**


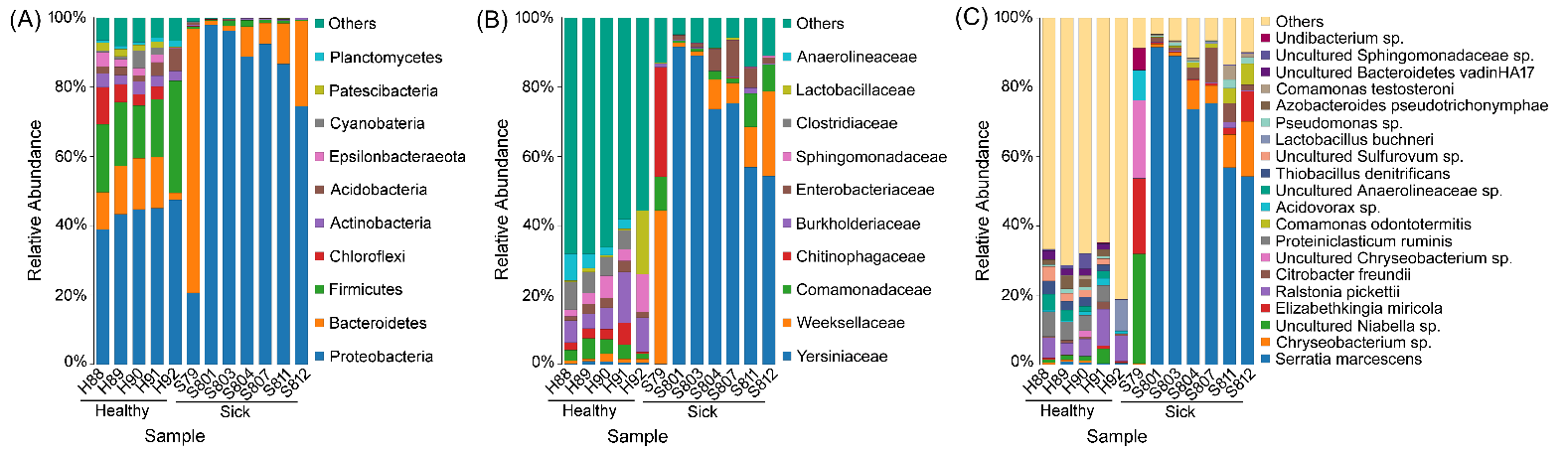


**Figure S6** Composition of skin bacteria of crocodile lizards at the Gandong station in 2020 at the phylum (A), family (B), and species (C) levels.


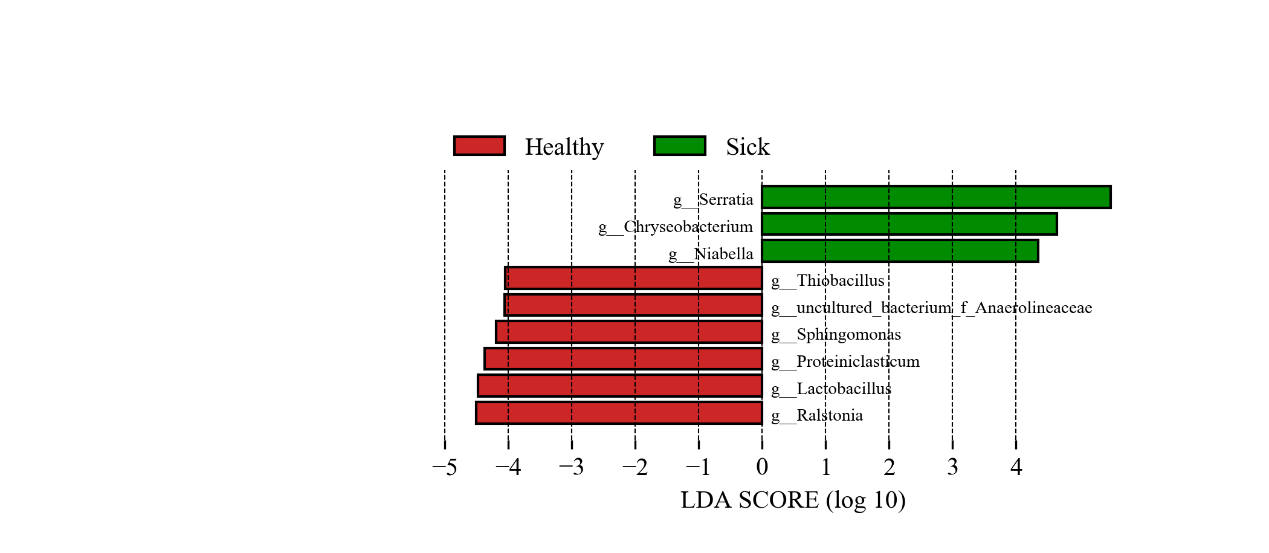


**Figure S7** Bacterial genera with significant abundance difference between the ulcerated and healthy skin samples. The highlighted taxa are significantly enriched in the group that corresponds to each color.


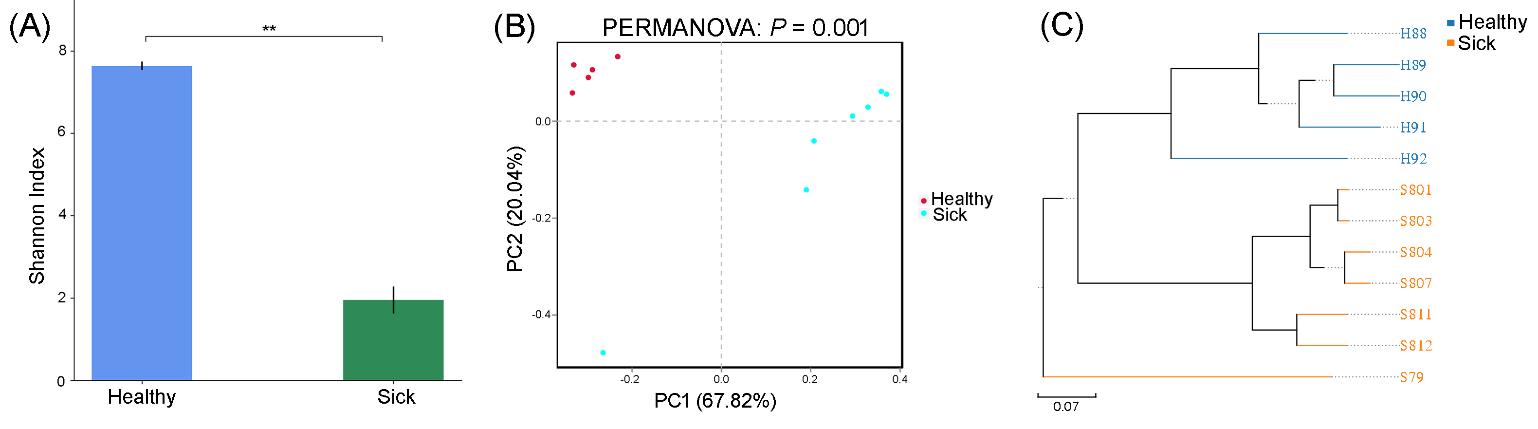


**Figure S8** Community diversity of the skin bacterial microbiome of crocodile lizards at the Gandong station in 2020. (A) Alpha diversity indicated by Shannon index. **, *P* < 0.05 according to Wilcoxon test. (B) Beta diversity indicated by principal coordinate analysis (PCoA) and based on the weighted UniFrac distance matrix. Numbers inside the parenthesis in the axis label show the percentage variation explained by each PC. *P* value of PERMANOVA test is noted at the top of PCoA plot. (C) Beta diversity indicated by the UPGMA cluster and based on the weighted UniFrac distance matrix.


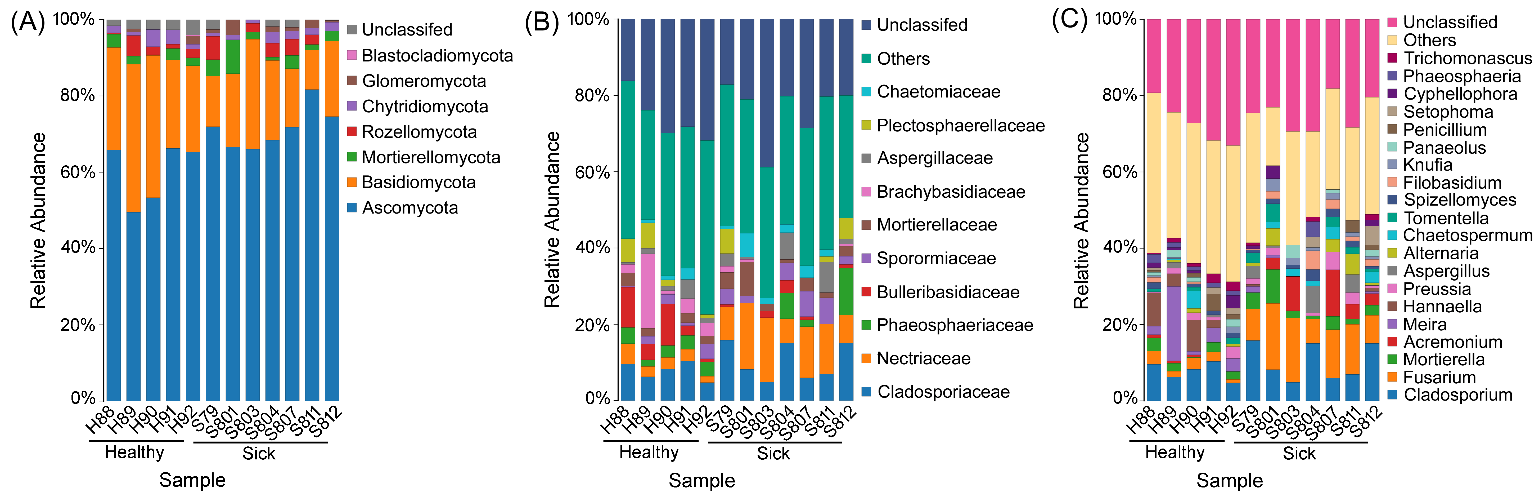


**Figure S9** Composition of cutaneous fungi of crocodile lizards at the Gandong station in 2020at the phylum (A), family (B), and genus (C) levels.


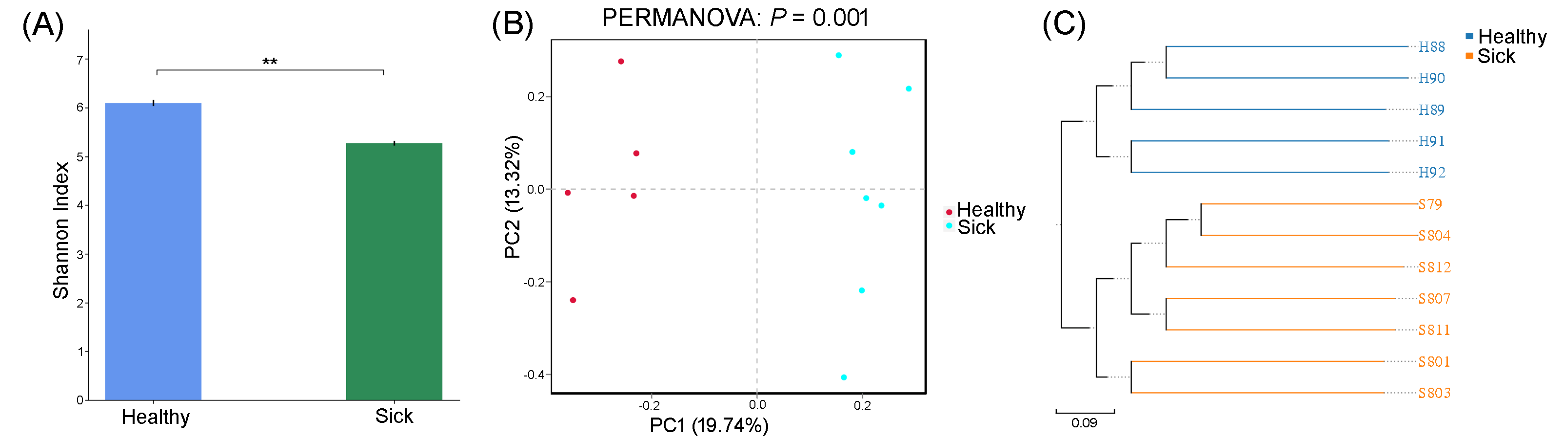


**Figure S10** Community diversity of the skin mycobiome of crocodile lizards at the Gandong station in 2020. (A) Alpha diversity indicated by Shannon index. **, *P* < 0.05 according to Wilcoxon test. (B) Beta diversity indicated by principal coordinate analysis (PCoA) and based on the bray curtis distance matrix. Numbers inside the parenthesis in the axis label show the percentage variation explained by each PC. *P* value of PERMANOVA test is noted at the top of PCoA plot. (C) Beta diversity indicated by the UPGMA cluster based on the bray curtis distance matrix.
